# Supplementary material for: Insights into Some Onygenalean Fungi from Freshwater Sediments in Spain and Description of Novel Taxa
Source: J Fungi (Basel). 2023 Nov 22;9(12):1129. doi: 10.3390/jof9121129 (PMC10744713; doi:10.3390/jof9121129)
Supplement: Supplementary file 1 [file jof-09-01129-s001.zip › jof-2711798-supplementary.pdf]

Table S1. Strain information and GenBank/EMBL accession numbers of the species included in the phylogenies of this study.

Species	Strain number[1]	Substrate	Country	Accession Number[2]				Reference
				ITS	LSU	*tub*2	*rpb*2	
Ascosphaera apis	CBS 252.32	*Apis mellifera*	Copenhagen, Denmark	-	AY004344	-	-	[104]
	C.A.A Wynns 5004 C	Pollen of *Megachile rotunda*	USA	NR137060	NG058557	-	-	[105]
Albidomyces albicans	CBS 151.65 T(type of *Arachniotus albicans*)	Pasture soil	UK	MH858527	NG057151	-	-	[106]
	CBS 808.71	Unknown	The Netherlands	MH860366	-	-	-	[106]
	FMR 17576	River sediments	Spain	ON720226	ON720765	-	-	This study
	FMR 18029	River sediments	Spain	ON721307	ON720780	-	-	This study
	FMR 18030	River sediments	Spain	ON720223	ON720762	-	-	This study
	FMR 18031	River sediments	Spain	ON720231	ON720770	-	-	This study
	FMR 18033	River sediments	Spain	ON720228	ON720767	-	-	This study
	FMR 18697	River sediments	Spain	OP373730	OP373734	-	-	This study
	FMR 18698	River sediments	Spain	ON720224	ON720763	-	-	This study
	FMR 18699	River sediments	Spain	OP373731	OP373735	-	-	This study
	FMR 19026	River sediments	Spain	ON720229	ON720768	-	-	This study
	FMR 19027	River sediments	Spain	ON720225	ON720764	-	-	This study
	FMR 19028	River sediments	Spain	ON720227	ON720766	-	-	This study
	FMR 19029	River sediments	Spain	ON720230	ON720769	-	-	This study
Amauroascopsis perforata	FMR 3882T	chicken yard soil sample	Burundi	AJ390377	-	-	-	[107]
Amauroascus niger	CBS 114.61T	Soil	California, USA	MH869547	AY176706	-	-	[106]
Amauroascus purpureus	IFO 32622T	Soil	Japan	AJ271564	AY176707	-	-	[108]
Amauroascus volatilis-patellis	CBS 249.72T	Soil	Utah, USA	MH860467	MH872189	-	-	[106]
Aphanoascus crassitunicatus	CBS 167.78T	Human toenail	France	OW987740	MH872884	-	-	[106]
	IHEM 23821	Human fingernail	Congo	OW987093	-	-	-	Only on database
	IHEM 23817	Monoascospore isolate from RV 35694	Belgium	OW987091	-	-	-	Only on database
	FMR 18700	River sediments	Spain	ON720203	ON720742	-	-	This study
	FMR 18701	River sediments	Spain	ON720204	ON720743	-	-	This study
	FMR 18702	River sediments	Spain	ON721311	ON720784	-	-	This study
Aphanoascus fulvescens	CBS 115955T	Human nail of toe	The Netherlands	KT155718	KT155038	-	-	Only on database
	CBS 743.68	Unknown	Switzerland	MH859219	MH870944	-	-	[106]
	NBRC 30411	Unknown	Japan	JN943432	JN941547	-	-	[109]
	CBS 741.68	Unknown	Germany	MH859217	MH870942	-	-	[106]
	FMR 18235	River sediments	Spain	ON720208	ON720747	-	-	This study

	FMR 18236	River sediments	Spain	ON720210	ON720749	-	-	This study
	FMR 18241	River sediments	Spain	ON721308	ON720781	-	-	This study
	FMR 18242	River sediments	Spain	ON720211	ON720750	-	-	This study
	FMR 18243	River sediments	Spain	ON942224	ON942226	-	-	This study
	FMR 18704	River sediments	Spain	ON720207	ON720746	-	-	This study
	FMR 19018	River sediments	Spain	ON720212	ON720751	-	-	This study
	FMR 19019	River sediments	Spain	ON720209	ON720748	-	-	This study
	FMR 19020	River sediments	Spain	ON721312	ON720785	-	-	This study
	FMR 19021	River sediments	Spain	ON720205	ON720744	-	-	This study
	FMR 19022	River sediments	Spain	ON720206	ON720745	-	-	This study
Aphanoascus mephitalis	ATCC 22144	Wolf dung	Ontario, Canada	MH859941	AY176725	-	-	[106]
Aphanoascus reticulisporus	CBS 392.67[T]	Unknown	New Zealand	MH859002	MH870704	-	-	[106]
	ChryCu	Human invasive pulmonary infection	Unknown	KJ808704	-	-	-	[110]
	NBRC 32372	Unknown	New Zealand	JN943434	JN941549	-	-	[109]
	UAMH 4320	Wool of *Sicistae betulinae*	USSR	AJ007841	-	-	-	[36]
	FMR 18004	River sediments	Spain	ON720200	ON720739	-	-	This study
	FMR 19012	River sediments	Spain	ON720201	ON720740	-	-	This study
	FMR 19013	River sediments	Spain	ON720202	ON720741	-	-	This study
	FMR 19033	River sediments	Spain	OP373732	OP373736	-	-	This study
Aphanoascus verrucosus	CBS 171.72; MUCL 10068[T] (type of *Chrysosporium tropicum*)	Woollen overcoat	Solomon Islands	MH858134	AY176731	-	-	[106]
Apinisia graminicola	CBS 721.68[T]	On rooting *Poaceae*	UK	-	NG056945	-	-	[111]
Apinisia racovitzae	CBS 156.77[T]	Skin lesion	USA	MZ435253	AB040696	-	-	[8]
Arachniotus verruculosus	CBS 655.71	Soil	Utah, USA	NR145221	AB040684	-	-	Only on database
Arachnomyces bostrychodes	CBS 146926	Human scalp	Texas, USA	LR701765	LR701766	-	-	[51]
Arachnomyces pilosus	CBS 250.93[T]	River sediments	Catalonia, Spain	MF572320	MF572325	-	-	[46]
Arachnotheca glomerata	CBS 348.71[IT]	Soil	Central Africa	-	NG056931	-	-	[106]
	CBS 349.71	Soil	Central Africa	MH860158	MH871926	-	-	[106]
Ascosphaera subglobosa	C.A.A. Wynns 5004 C	Pollen of *Megachile rotundata*	Utah, USA	NR137060	HQ540517	-	-	[105]
Arthroderma curreyi	CBS 353.66[T]	Unknown	UK	MH858822	MH870459	-	-	[106]
	FMR 19038	River sediments	Spain	ON720238	ON720777	-	-	This study
Arthroderma onychocola	CBS 132920[T]	Human nail	Prague, Czech Republic	KT155794	KT155124	-	-	Only on database
Auxarthronopsis bandhavgarhensis	NFCCI 2185[T]	Soil	Bandhavgarh, India	HQ164436	NG057012	-	-	[15]

Auxarthronopsis guizhouensis	CGMCC 3.17910^{T}	Air	Guizhou, China	KU746668	KU746714	-	-	[112]
Blastomyces percursus	CBS 139878^{T}	From human	South Africa	NR153647	KY195971	-	-	[114]
Blastomyces dermatitidis	CBS 674.68 ^{T}	Unknown	USA	MH859201	MH870922	-	-	[106]
Canomyces reticulatus	MCC 1486^{T}	Soil	Maharashtra, India	MK340501	MK340502	-	-	[15]
Chrysosporium carmichaelii	CBS 643.79^{T}	Unknown	Michigan, USA	NR077133	NG058857	-	-	[113]
Chrysosporium chiropterorum	MUCL 45495^{T}	Fur of bat	France	AM949570	AM949570	-	-	[114]
Chrysosporium lobatum	CBS 275.77	On hair of *Mus musculus*	Russia	-	KT155192	-	-	Only on database
Chrysosporium pallidum	CGMCC 3.19575^{T}	Dung	Guangxi, China	NR172829	NG075267	-	-	[83]
Chrysosporium sulfureum	CBS 634.79	Cheese rind	Switzerland	KT155953	KT155298	-	-	Only on database
Chrysosporium undulatum	CBS 964.97^{T}	River sediments	Spain	NR157455	NG063939	-	-	[36], and on database
Chrysosporium vallenarense	CBS 627.83^{T}	Semi-desert	Chile	MH861669	NG063937	-	-	[106]
Ctenomyces serratus	CBS 187.61^{T}	On rotting feathers of Aves	Germany	MH858017	MH869579	-	-	[106]
Currahmyces indicus	MCC 1548^{T}	Hen resting area	Maharashtra, India	MK340498	MK340499	-	-	[15]
Currahmyces sparsispora	CBS 146929^{T}	Human sputum	Florida, USA	LR723272	LR723273	-	-	[51]
Emergomyces crescens	CBS 177.60 ^{T}	Unknown	Norway	MH857947	MH869492	-	-	[106]
Emergomyces pasteurianus	CBS 101236^{T}	Human cutaneous lesion	Italy	-	NG070831	-	-	[14]
Emmonsiellopsis coralliformis	FMR 4024^{T}	River sediments	Spain	NR153996	NG059238	-	-	[37]
Emmonsiellopsis terrestris	UAMH 2304^{T}	Soil	USA	AF038320	AF038320	-	-	[48]
Emmonsiellopsis tuberculata	FMR 17582^{T}	River sediments	Spain	LR598892	LR598891	-	-	[38]
Emydomyces testavorans	ATCC TSD-145 ^{T}	Shell lesions of a turtle	USA	NR160604	NG066406	-	-	[95]
Epidermophyton floccosum	CBS 230.76^{T}	Human	Unknown	KT155837	KT155176	-	-	[115]
Guarromyces ceretanicus	CBS 269.89^{T}	Unknown	Unknown	NR154051	NG058484	-	-	[116]
Gymnoascoideus petalosporus	CBS 252.72	Unknown	Unknown	-	AB040685	-	-	[117]
	FMR 19036	River sediments	Spain	ON720236	ON720775	-	-	This study
Gymnoascus reesii	CBS 410.72	Soil	California, USA	MH860507	MH872224	-	-	[106]
Helicoarthrosporum mellicola	CBS 143838^{T}	Honey	Granada, Spain	LR761645	LT906535	-	-	[51]
	FMR 15673	Honey	Valencia, Spain	LR761646	LT978462	-	-	[51]
Helicocarpus griseus	CBS 128.88^{T}	Unknown	Algeria	MH862124	MH873814	-	-	[106]
Histoplasma capsulatum	UAMH 3536^{T}	Woman biopsy of right lung	Alberta, Canada	AF038354	AF038354	-	-	[48]
Leucothecium emdenii	CBS 576.73^{T}	Soil	The Netherlands	-	NG057812	-	-	[118]
	CBS 370.92	Marine sediments	Spain	AB213434	HG004546	-	-	[87]
	FMR 18703	River sediments	Spain	ON720239	ON720778	-	-	This study
Lophophyton gallinae	CBS 243.66	Infection in *Canis lupus*	Montana, USA	KT155842	KT155181			[115]
Malbranchea albolutea	CBS 125.77^{T}	Soil	Utah, USA	MH861039	MH872808	-	-	[106]

	FMR 17679	Human BAL	Texas, USA	LR701834	LR701835	-	-	[51]
Malbranchea aurantiaca	CBS 127.77^T	Culture contaminant	Utah, USA	NR157447	AB040704	-	-	[117]
	FMR 17682	Animal skin lesion	Texas, USA	LR701826	LR701827	-	-	[51]
	FMR 17688	Animal	California, USA	LR701824	LR701825	-	-	[51]
Malbranchea californiensis	ATCC 15600^T	Dung of pack rat	California, USA	MH858121	MH858121	-	-	[106]
Malbranchea chinensis	CGMCC 3.19572^T	Soil of karst cave	Guanxi, China	NR172823	MK328981	MK336102	-	[83]
	FMR 18267	River sediments	Spain	ON720190	ON720729	OP425706	OP425715	This study
Malbranchea chlamydospora	RV 24809 ^{T}	Unknown	Unknown	AJ271425	-	-	-	[108]
Malbranchea chrysosporoidea	CBS 128.77^T	Soil	Arizona, USA	AB361632	AB359413	-	-	Only on database
Malbranchea cinnamomea	CBS 343.55	Old straw in poultry farm	The Netherlands	MH857506	KT155221	-	-	[106]
Malbranchea circinata	CBS 129.77^T	Soil	Utah, USA	MN627784	MN627782	-	-	[119]
Malbranchea compacta	CBS 200.64^T	Unknown	California, USA	MH858415	MH870043	-	-	[106]
Malbranchea concentrica	CBS 112861^T	Unknown	Unknown	NR111089	-	-	-	[108]
Malbranchea conjugata	CBS 247.58	Soil	Arizona, USA	NR121475	HF545313	HE974414	HE974413	[113]
	FMR 17697	Human BAL	Texas, USA	LR701830	LR701831	-	-	[51]
	FMR 17699	Human lung tissue	Florida, USA	LR701828	LR701829	-	-	[51]
Malbranchea dendritica	CBS 131.77^T	Soil	Utah, USA	AY177310	AB359416	-	-	[120]
Malbranchea echinulata	FMR 17906^T	River sediments	Spain	ON720198	ON720737	OP425705	-	This study
Malbranchea filamentosa	CBS 581.82^T	Soil	Argentina	NR111136	AB359417	-	-	[113]
Malbranchea flava	CBS 132.77^T	Soil	California, USA	AB361633	AB359418	-	-	Only on database
Malbranchea flocciformis	CBS 133.77^T	Saline soil	France	AB361634	AB359420	-	-	Only on database
	FMR 17698	Human skin	Texas, USA	LR701822	LR701823	-	-	[51]
Malbranchea fulva	CBS 135.77^T	Air	Utah, USA	NR157444	AB359422	-	-	Only on database
Malbranchea guanxiense	CGMCC 3.19634^T	Soil of karst cave	Guanxi, China	MK329080	MK328985	MK336106	-	[85]
Malbranchea gymnoascoides	CBS 146930^T	Human BAL	Texas, USA	LR701757	LR701758	-	-	[51]
Malbranchea irregularis	FMR 19016^T	River sediments	Spain	ON720191	ON720730	OP425710	OP425719	This study
	FMR 19017	River sediments	Spain	ON720192	ON720731	OP425713	OP425722	This study
	FMR 19030	River sediments	Spain	ON720193	ON720732	OP425712	OP425721	This study
	FMR 19015	River sediments	Spain	ON720194	ON720733	OP425711	OP425720	This study
Malbranchea kuehnii	CBS 539.72^T	Dung	Unknown	NR103573	NG056928	-	-	[113]
Malbranchea sinuata	FMR 18266^T	River sediments	Spain	ON720195	ON720734	OP425704	OP425714	This study
Malbranchea longispora	FMR 12768^T	Soil	Beija, Portugal	HG326873	HG326874	-	-	[121]
Malbranchea multiseptata	CBS 146931^T	Human BAL	Texas, USA	LR701759	LR701760	-	-	[51]

Malbranchea ostraviensis	CCF 4241[T]	Human Fingernails	Czech Republic	NR121474	-	HE974417	HE974411	[113]
	FMR 18693	River sediments	Spain	ON720199	ON720738	OP425707	OP425716	This study
Malbranchea pseudoauxarthron	CBS 657.71[T]	Unknown	Unknown	MH860293	-	-	-	[106]
Malbranchea pseudoreticulata	UAMH 3117[T]	Lizard dung	Mexico	NR111111	-	-	-	[108]
Malbranchea pulchella	CBS 202.38	Unknown	Italy	AB361638	AB359426	-	-	Only on database
Malbranchea reticulata	CBS 201.64[T]	Wood from greenhouse flat	California, USA	OW986827	OW986827	-	-	Only on data base
	FMR 18696	River sediments	Spain	ON721310	ON720783	-	-	This study
Malbranchea setosa	CBS 198.92[T]	Soil	Congo	KT155638	-	-	-	Only on database
Malbranchea stricta	CBS 146932[T]	Human nail	Florida, USA	LR701638	LR701639	-	-	[51]
Malbranchea thaxteri	CBS 248.58[T]	Opossum dung	Haiti	NR111138	-	HE974416	HE974412	[113]
Malbranchea umbrina	CBS 105.09[T]	Soil	UK	MH854591	MH866116	HE974415	HE974407	[106]
	CBS 226.58	Unknown	Unknown	MH857765	MH869296	-	-	[106]
	FMR 17693	Human nail	Washington DC, USA	LR701820	LR701821	-	-	[51]
	FMR 17899	River sediments	Spain	ON721306	ON720779	-	-	This study
	FMR 18695	River sediments	Spain	ON720196	ON720735	OP425709	OP425718	This study
	FMR 18766	River sediments	Spain	ON720197	ON720736	OP425708	OP425717	This study
Malbranchea zuffiana	CBS 219.58[T]	Prairie dog lung	Texas, USA	MH869293	AY176712	-	-	[106]
Mallochia reticulata	CBS 392.61[T]	Unknown	Honduras	MH858097	MH869667	-	-	[108]
Microsporum audouinii	CBS 545.93[T]	Human	France	NR144883	NG069298	-	-	[122]
Myotsia cremea	CBS 141864[T]	Bat droppings	Czech Republic	NG088058	NG088058	-	-	[123]
Myriodontium keratinophilum	CBS 947.73[T]	Soil	Italy	NR157454	NG063938	-	-	Only on database
	CBS 256.81	Unknown	The Netherlands	MH861337	MH873097	-	-	[106]
	IHEM 19171	Persian cat	Belgium	OW985860	-	-	-	Only on database
	FMR 17624	River sediments	Spain	ON720220	ON720759	-	-	This study
	FMR 18244	River sediments	Spain	ON720222	ON720761	-	-	This study
	FMR 18245	River sediments	Spain	ON720213	ON720752	-	-	This study
	FMR 18246	River sediments	Spain	ON720221	ON720760	-	-	This study
	FMR 18247	River sediments	Spain	ON721309	ON720782	-	-	This study
	FMR 18248	River sediments	Spain	ON720215	ON720754	-	-	This study
	FMR 18249	River sediments	Spain	ON720216	ON720755	-	-	This study
	FMR 18250	River sediments	Spain	ON720218	ON720757	-	-	This study
	FMR 18251	River sediments	Spain	ON720217	ON720756	-	-	This study
	FMR 18257	River sediments	Spain	ON720214	ON720753	-	-	This study

	FMR 18258	River sediments	Spain	ON720219	ON720758	-	-	This study
	FMR 19031	River sediments	Spain	OP070725	OP077087	-	-	This study
Nannizzia incurvata	CBS 174.64[T]	Human	UK	MH858406	MH870033	-	-	[106]
Nannizziopsis guarroi	CBS 124553[T]	Iguana skin	Spain	MH863384	MH874904	-	-	[106]
Nannizziopsis vriesii	CBS 407.71[T]	Ameiva (lizard) skin and lung	The Netherlands	AJ131687	AY176715	-	-	[111]
Neoarthropsis hispanica	CBS 351.92[T] (type of *Arthropsis hispanica*)	Marine sediments	Spain	HE965758	HE965759	-	-	[81]
	UTHSC 09-3174	Bronchial wash	USA	HE965756	HE965757	-	-	[81]
	FMR 12113	Nails	USA	KP131547	-	-	-	[124]
	FMR 18694	River sediments	Spain	ON720234	ON720773	-	-	This study
	FMR 19034	River sediments	Spain	ON720232	ON720771	-	-	This study
	FMR 19035	River sediments	Spain	ON720233	ON720772	-	-	This study
Neoarthropsis sexualis	FMR 19025[T]	River sediments	Spain	ON720235	ON720774	-	-	This study
Neogymnomyces demonbreunii	ATCC 18394	Unknown	Missouri, USA	AJ315842	AY176716	-	-	[106]
Onygena corvina	CBS 152.73[NT]	Unknown	The Netherlands	OR167982	MH878305	-	-	[106]
Onygena equina	CBS 947.70[NT]	Unknown	Unknown	MZ159461	AB075356	-	-	[49]
Paracoccidioides brasiliensis	UAMH 8037[T]	Human lung biopsy	Alberta, Canada	AF038360	AF038360	-	-	[48]
Paraphyton cookei	CBS 228.58[T]	Unknown	Unknown	NR155665	NG058188	-	-	Only on database
Polytolypa hystricis	UAMH 7299[T]	From dung	Canada	NR111161	NG042396	-	-	[113]
Pseudoamaurascopsis spiralis	FMR 19014[T]	River sediments	Spain	ON720237	ON720776	-	-	This study
Pseudoarthropsis cirrhata	CBS 628.83[T]	Wall sample	The Netherlands	-	NG060792	-	-	[87]
Pseudoarthropsis crassispora	CBS 146928	Human BAL	Minnesota, USA	LR701763	LR701764	-	-	[51]
Pseudomalbranchea gemmata	CBS 146933[T]	Human BAL	Florida, USA	LR701761	LR701762	-	-	[51]
Pseudospiromastix tentaculata	CBS 184.92[T]	Soil	Hiram, Somalia	AY527406	LN867603	-	-	[50]
Renispora flavissima	ATCC 38503[T]	Soil	Kansa, USA	AJ390392	NG059414	-	-	[111]
Shanorella spirotricha	CBS 304.56[T]	On feathers of dead *Aves*	California, USA	MH857651	MH869194	-	-	[106]
Sigleria amenda	CBS 138257[T]	House dust	Mexico	KP119656	KP119643	-	-	[125]
Sigleria carmichaelii	CBS 138264[T]	House dust	Micronesia	KP119626	KP119638	-	-	[125]
Spiromastigoides alatospora	CBS 457.73[T]	Unknown	India	MH860740	MH872453	-	-	[106]
Spiromastigoides albida	CBS 139510[T]	Human lung biopsy	Texas, USA	LN867606	LN867602	-	-	[126]
Spiromastigoides asexualis	CBS 136728[T]	From shephered dog	USA	KJ880032	LN867603	-	-	[127]
Spiromastigoides curvata	JCM 11275[T]	Contaminant of a *H. capsulatum* strain	Mexico	KP119631	KP119644	-	-	[125]
Spiromastigoides frutex	CBS 138266[T]	House dust, rental studio	Nayarit, Mexico	KP119632	KP119645	-	-	[125]
Spiromastigoides geomyces	CBS 146934	Human skin foot	Illinois, USA	LR701767	LR701768	-	-	[51]
Spiromastigoides gypsea	CBS 134.77[T]	Soil	California, USA	KT155798	NG063935	-	-	[51]

Spiromastigoides kosraensis	CBS 138267[T]	House dust	Kosrae, Micronesia	KP119633	KP119646	-	-	[125]
Spiromastigoides pyramidalis	CBS 138269[T]	House dust	Australia	KP119636	KP119649	-	-	[125]
Spiromastigoides sugiyamae	JCM 11276[T]	Soil	Japan	LN867608	AB040680	-	-	[126]
Spiromastigoides warcupii	CBS 576.63[T]	Soil	Australia	LN867609	AB040679	-	-	[117]
Strongyloarthrosporum catenulatum	CBS 143841[T]	Honey	Toledo	LR760230	LT906534	-	-	[51]
Trichophyton bullosum	CBS 363.35[T]	Unknown	Unknown	NR144895	NG058191	-	-	[129]
Trichophyton tonsurans	CBS 496.48[T]	Unknown	France	MH856446	MH867992	-	-	[106]
Uncinocarpus reesii	ATCC 34533	Feather	Australia	MH861035	AY176724	-	-	[106]

[1]ATCC: American Type Culture Collection, USA; CBS: Culture collection of the Westerdijk Fungal Biodiversity Institute, Utrecht, the Netherlands; CCF: Culture Collection of Fungi, Faculty of Science, Charles University, Benátská, Czech Republic. CGMCC: China General Microbiological Culture Collection Centre, Institute of Microbiology, Chinese Academy of Sciences, Beijing, China; ChryCU: C.A.A. Wynns; FMR: Facultat de Medicina i Ciències de la Salut, Reus, Spain; IFO: Institute for Fermentation, Osaka, Japan; JCM: Japan Collection of Microorganisms, Institute of Physical and Chemical Research, Wako, Japan; MCC: Microbial Culture Collection, Pune, India; NFCCI: National Fungal Culture Collection of India, New Dehli, India; NRRL: Agricultural Research Service Culture Collection (NRRL - Northern Regional Research Laboratory) Database, Lubbock, Texas, USA; RV: Collection of Leptospira Strains, Higher Institute of Sanita, Roma-Nomentano, Italy; UAMH: Center for Global Microfungal Biodiversity, Dalla Lana School of Public Health, University of Toronto, Toronto, Canada. [2]ITS: Internal transcribed spacer region of the rDNA and 5.8S region; LSU: Large subunit region of the rDNA; *tub*2: partial β-tubulin gene; *rpb2*: the DNA dependent RNA polymerase II largest subunit. Known species identified in this study and taxonomic novelties proposed are in bold. [T] indicates ex-type strains, [IT] indicates ex-isotype strains, [NT] indicates ex-neotype strains.

REFERENCES

8. Kandemir H, Dukik K, de Melo Teixeira M, Stielow JB, Delma FZ, Al-Hatmi A, Ahmed SA, Ilkit M, De Hoog GS. 2022. Phylogenetic and ecological reevaluation of the order *Onygenales*. *Fungal Diversity* 1–72. https://doi.org/10.1007/s13225-022-00506-z.

14. Dukik K, Muñoz JF, Jiang Y, Feng P, Sigler L, Stielow JB, de Hoog S. 2017. Novel taxa of thermally dimorphic systemic pathogens in the *Ajellomycetaceae* (*Onygenales*). *Mycoses* 60: 296–309. https://doi.org/10.1111/myc.12601.

15. Sharma R, Shouche YS. 2020. Diversity of onygenalean fungi in keratin-rich habitats of Maharashtra (India) and description of three novel taxa. *Mycopathologia* 185: 67–85. https://doi.org/10.1007/s11046-019-00346-7.

36. Vidal P, Ulfig K, Valmaseda M, Guarro J. 1999. Studies on keratinophilic fungi. XI. *Chrysosporium undulatum* sp. nov. *Antonie van Leeuwenhoek 75*: 171–182. https://doi.org/10.1023/A:1001734015104.

37. Marin-Felix Y, Stchigel AM, Cano-Lira JF, Sanchis M, Mayayo E, Guarro J. 2015. Emmonsiellopsis, a new genus related to the thermally dimorphic fungi of the family Ajellomycetaceae. *Mycoses* 58: 451–460. https://doi.org/10.1111/myc.12336.

38. Crous PW, Wingfield MJ, Lombard L, Roets F, Swart WJ, Alvarado P, Carnegie AJ, Moreno G, Luangsa-ard J, Thangavel R, et al. 2019. Fungal Planet description sheets: 951–1041. *Persoonia: Molecular Phylogeny and Evolution of Fungi* 43: 223–425. https://doi.org/10.3767/persoonia.2019.43.06.

46. Sun YGZ, Chen AJ, Houbraken J. 2019. Phylogeny and a new species of the genus Arachnomyces (Arachnomycetaceae). *Phytotaxa* 394: 089–097. https://doi.org/10.11646/phytotaxa.394.1.6b.

48. Peterson SW, Sigler L. 1998. Molecular genetic variation in *Emmonsia crescens* and *Emmonsia parva*, etiologic agents of adiaspiromycosis, and their phylogenetic relationship to *Blastomyces dermatitidis* (*Ajellomyces dermatitidis*) and other systemic fungal pathogens. *Journal of Clinical Microbiology* 36: 2918–2925. https://doi.org/10.1128/jcm.36.10.2918-2925.1998.

50. Untereiner WA, Scott JA, Naveau FA, Sigler L, Bachewich J, Angus A. 2004. The *Ajellomycetaceae*, a new family of vertebrate-associated *Onygenales*. *Mycologia* 96: 812–821. https://doi.org/10.1080/15572536.2005.11832928.

51. Rodríguez-Andrade E, Cano-Lira JF, Wiederhold N, Perez-Cantero, A, Guarro J, Stchigel AM. 2021. A revision of malbranchea-like fungi from clinical specimens in the United States of America reveals unexpected novelty. *IMA Fungus* 12. https://doi.org/10.1186/s43008-021-00075-x.

81. Giraldo A, Sutton DA, Gené J, Fothergill AW, Cano J, Guarro J. 2013. Rare arthroconidial fungi in clinical samples: *Scytalidium cuboideum* and *Arthropsis hispanica*. *Mycopathologia* 175: 115–121. https://doi.org/10.1007/s11046-012-9590-3.
83. Zhang ZF, Zhou SY, Eurwilaichitr L, Ingsriswang S, Raza M, Chen Q, Zhao P, Liu F, Cai L. 2021. Culturable mycobiota from Karst caves in China II, with descriptions of 33 new species. *Fungal Diversity* 106: 29–136. https://doi.org/10.1007/s13225-020-00453-7.
87. Giraldo A, Gené J, Sutton DA, Madrid H, Cano J, Crous PW, Guarro J. 2014. Phylogenetic circumscription of *Arthrographis* (*Eremomycetaceae*, *Dothideomycetes*). *Persoonia-Molecular Phylogeny and Evolution of Fungi* 32: 102–114. http://dx.doi.org/10.3767/003158514X680207.
95. Woodburn DB, Miller AN, Allender MC, Maddox CW, Terio KA. 2019. *Emydomyces testavorans*, a new genus and species of onygenalean fungus isolated from shell lesions of freshwater aquatic turtles. *Journal of Clinical Microbiology* 57: e00628-18. https://doi.org/10.1128/JCM.00628-18.
104. Lumbsch HT, Lindemuth R, Schmitt I. 2000. Evolution of filamentous ascomycetes inferred from LSU rDNA sequence data. *Plant Biology* 2: 525–529. https://doi.org/10.1055/s-2000-7472.
105. Wynns AA, Jensen AB, Eilenberg J, James R. 2012. *Ascosphaera subglobosa*, a new spore cyst fungus from North America associated with the solitary bee *Megachile rotundata*. *Mycologia* 104: 108–114. https://doi.org/10.3852/10-047.
106. Vu D, Groenewald M, De Vries M, Gehrmann T, Stielow B, Eberhardt U, Al-Hatmi A, Groenewald JZ, Cardinali G, Houbraken J, Boekhout T, Crous PW, Robert V, Verkley GJM. 2019. Large-scale generation and analysis of filamentous fungal DNA barcodes boosts coverage for kingdom fungi and reveals thresholds for fungal species and higher taxon delimitation. *Studies in Mycology* 92: 135–154. https://doi.org/10.1016/j.simyco.2018.05.001.
107. Vidal P, Vinuesa MDLA, Sánchez-Puelles JM, Guarro J. 2000. Phylogeny of the anamorphic genus *Chrysosporium* and related taxa based on rDNA internal transcribed spacer sequences. *Revista Iberoamericana de Micology* 17: 22–29.
108. Solé M, Cano J, Guarro J. 2002. Molecular taxonomy of *Amauroascus, Auxarthron* and other related genera of the *Onygenales*. *Mycological Research* 106: 388–396.
109. Schoch CL, Seifert KA, Huhndorf S, Robert V, Spouge JL, Levesque CA, Chen W, White MM. 2012. Nuclear ribosomal internal transcribed spacer (ITS) region as a universal DNA barcode marker for Fungi. *Proceedings of the National Academy of Sciences* 109: 6241–6246. https://doi.org/10.1073/pnas.1117018109.
110. Suankratay C, Dhissayakamol O, Uaprasert N, Chindamporn A. 2015. Invasive pulmonary infection caused by *Chrysosporium articulatum*: the first case report. *Mycoses* 58: 1–3. https://doi.org/10.1111/myc.12270.
111. Untereiner WA, Scott JA, Naveau FA, Bachewich J. 2002. Phylogeny of *Ajellomyces*, *Polytolypa* and *Spiromastix* (*Onygenaceae*) inferred from rDNA sequence and non-molecular data. *Studies in Mycology* 47: 25–35.
112. Zhang ZF, Liu F, Zhou X, Liu XZ, Liu SJ, Cai L. 2017. Culturable mycobiota from Karst caves in China, with descriptions of 20 new species. *Persoonia-Molecular Phylogeny and Evolution of Fungi* 39: 1–31. https://doi.org/10.3767/persoonia.2017.39.01.
113. Schoch CL, Robbertse B, Robert V, Vu D, Cardinali G, Irinyi L, Meyer W, Nilsson RH, Hughes K, Miller AN, et al. 2014. Finding needles in haystacks: linking scientific names, reference specimens and molecular data for Fungi. *Database*: 2014. https://doi.org/10.1093/database/bau061.
114. Nováková A, Kolařík M. 2010. *Chrysosporium speluncarum*, a new species resembling *Ajellomyces capsulatus*, obtained from bat guano in caves of temperate Europe. *Mycological Progress 9*: 253–260. https://doi.org/0.1007/s11557-009-0634-0.
115. Stielow JB, Lévesque CA, Seifert KA, Meyer W, Iriny L, Smits D, Renfurm R, Verkley GJ, Groenewald M, Chaduli D, et al. 2015. One fungus, which genes? Development and assessment of universal primers for potential secondary fungal DNA barcodes. Persoonia 35: 242-63. https://doi..org/10.3767/003158515X689135.
116. Brasch J, Gräser Y. 2005. Trichophyton eboreum sp. nov. isolated from human skin. *Journal of Clinical Microbiology* 43: 5230–5237. https://doi.org/10.1128/jcm.43.10.5230-5237.2005.
117. Sugiyama M, Mikawa T. 2001. Phylogenetic analysis of the non-pathogenic genus *Spiromastix* (*Onygenaceae*) and related onygenalean taxa based on large subunit ribosomal DNA sequences. *Mycoscience* 42: 413–421. https://doi.org/10.1007/BF02464337.
118. Gueidan C, Villaseñor CR, De Hoog GS, Gorbushina AA, Untereiner WA, Lutzoni F. 2008. A rock-inhabiting ancestor for mutualistic and pathogen-rich fungal lineages. *Studies in Mycology* 61: 111–119. https://doi.org/10.3114/sim.2008.61.11.
119. Rangel-Grimaldo M, Macías-Rubalcava ML, González-Andrade M, Raja H, Figueroa M, Mata R. 2020. α-Glucosidase and protein tyrosine phosphatase 1B inhibitors from Malbranchea circinata. *Journal of Natural Products* 83: 675–683. https://doi.org/10.1021/acs.jnatprod.9b01108.

120. Sigler L, Hambleton S, Flis AL, Pare JA. 2002. *Auxarthron* teleomorphs for *Malbranchea filamentosa* and *Malbranchea albolutea* and relationships within *Auxarthron*. *Studies in Mycology* 47: 111–122.
121. Crous PW, Wingfield MJ, Guarro J, Cheewangkoon R, Van der Bank M, Swart WJ, Stchigel AM, Cano-Lira JF, Roux J, Madrid H, et al. 2013. Fungal Planet description sheets: 154–213. *Persoonia-Molecular Phylogeny and Evolution of Fungi* 31: 188–296. https://doi.org/10.3767/003158513X675925.
122. Gräser Y, El Fari M, Vilgalys R, Kuijpers AFA, De Hoog GS, Presber W, Tietz HJ. 1999. Phylogeny and taxonomy of the family Arthrodermataceae (dermatophytes) using sequence analysis of the ribosomal ITS region. *Medical Mycology* 37: 105–114. https://doi.org/10.1080/02681219980000171.
123. Crous PW, Wingfield MJ, Burgess TI, Hardy GSJ, Barber PA, Alvarado P, Barnes CW, Buchanan PK, Heykoop M, Moreno G, et al. 2017. Fungal Planet description sheets: 558–624. *Persoonia: Molecular Phylogeny and Evolution of Fungi* 38: 240. https://doi.org/10.3767/003158517X698941.
124. Irinyi L, Serena C, Garcia-Hermoso D, Arabatzis M, Desnos-Ollivier M, Vu D, Cardinali G, Arthur IA, Normand AC, Giraldo A, et al. 2015. International Society of Human and Animal Mycology (ISHAM)-ITS reference DNA barcoding database—the quality controlled standard tool for routine identification of human and animal pathogenic fungi. *Medical Mycology 53*: 313–337. https://doi.org/10.1093/mmy/myv008.
125. Hirooka Y, Tanney JB, Nguyen HD, Seifert KA. 2016. Xerotolerant fungi in house dust: taxonomy of *Spiromastix*, *Pseudospiromastix* and *Sigleria* gen. nov. in *Spiromastigaceae* (*Onygenales*, *Eurotiomycetes*). *Mycologia* 108: 135–156. https://doi.org/10.3852/15-065.
126. Stchigel AM, Sutton DA, Cano-Lira JF, Wiederhold N, Guarro J. 2017. New species *Spiromastigoides albida* from a lung biopsy. *Mycopathologia* 182: 967–978. https://doi.org/10.1007/s11046-017-0179-8.
127. Rizzo L, Sutton DA, Wiederhold NP, Thompson EH, Friedman R, Wickes BL, Cano-Lina JF, Stchigel AM, Guarro J. 2014. Isolation and characterisation of the fungus *Spiromastix asexualis* sp. nov. from discospondylitis in a German Shepherd dog, and review of *Spiromastix* with the proposal of the new order *Spiromastixales* (*Ascomycota*). *Mycoses* 57: 419–428. https://doi.org/10.1111/myc.12178.
128. Heidemann S, Monod M, Gräser Y. 2010. Signature polymorphisms in the internal transcribed spacer region relevant for the differentiation of zoophilic and anthropophilic strains of *Trichophyton interdigitale* and other species of *T. mentagrophytes* sensu lato. *British Journal of Dermatology* 162: 282–295. https://doi.org/10.1111/j.1365-2133.2009.09494.x.

Table S2: Environmental and biogeographical information contained in all ITS1/ITS2 sequences downloaded from GlobalFungi database included in our analysis (see Fig. 4).

Hypothetical taxa	Sequence	ITS[1]	Sample ID[2]	Primers	Longitude	Latitude	Sample type	ITS observed[3]	ITS total[4]	Freq R.[5]	Biome	MAT[6]	MAP[7]	Ph	Geographical Origin	Dominant plants
Albidomyces albicans	b1676d95bf4d8bef02e8d020daf54120	ITS1	48285	ITS1ngs/ITS4ngs	21.8955°	58.3267°	Soil	2	2564	0.7800	Grassland	7	581	NA	Estonia	*Platanthera chlorantha*
	0ce42d77acf661aba7f517be3cb04cd8	ITS1	44192	ITS9MUNngs/ITS4ngsUni	25.8776°	57.8607°	Soil	1	756	1.3227	Forest	5.8	660	NA	Estonia	NA
	61d71d93f69bcd47883fdec679f54917	ITS1	30649	ITS9MUNngs/ITS4ngsUni	15.4480°	50.1600°	Soil	2	8462	0.2363	Forest	9.1	614	NA	Czech Republic	NA
	fc7e4c63b3e83ce3aff00e23ad8f7ff8	ITS1	26926	ITS9MUNngs/ITS4ngsUni	24.1268°	59.3655°	Soil	2	3207	0.6236	Urban	6.1	583	NA	Estonia	NA
	a178fbfb8d85a2a178146e77563b404b	ITS1	22060	ITS9MUNngs/ITS4ngsUni	23.5513°	58.5621°	Soil	1	15100	0.0662	Forest	6.6	587	NA	Estonia	NA
	357f72f9698f744f4f56bbd7ffefc8ed	ITS1	39251	ITS9MUNngs/ITS4ngsUni	22.9943°	56.7230°	Soil	5	4890	1.0224	Forest	6.7	596	NA	Latvia	NA
	54e1a2cd6e91fbd67a7b79e3861bb0dd	ITS1	38105	ITS9MUNngs/ITS4ngsUni	21.8665°	58.2811°	Soil	1	3291	0.3038	Forest	7.1	569	NA	Estonia	NA
	678fddf2957f93724d265d14a821430e	ITS1	8733	ITS9MUNngs/ITS4ngsUni	23.6570°	58.7351°	Soil	2	6681	0.2993	Forest	6.5	607	NA	Estonia	NA
	1a354922ef3c78fb6658ee5e8d17a3b4|	ITS1	20390	ITS9MUNngs/ITS4ngsUni	22.8187°	58.2806°	Soil	2	4226	0.4732	Forest	6.9	575	NA	Estonia	NA
	553722bf58a45389bc3da13c70ac1ecf	ITS1	38221	ITS9MUNngs/ITS4ngsUni	24.5047°	59.3545°	Soil	1	3706	0.2698	Forest	5.9	650	NA	Estona	NA
	174926d143ff672eb12c4e029d299bd9	ITS2	17775	ITS1F/ITS4	9.34944	48.4969	Soil	6	6549	0.9161	Forest	7.5	974	NA	Germany	NA
	cdda15ba6b511e0df271275e2491f4eb	ITS2	10310	ITS9MUNngs/ITS4ngsUni	14.5822°	49.6684°	Soil	304	15920	19.0954	Forest	8.4	524	NA	Czech Republic	NA
	1a039be70e4b8b6370a4a1957d1a1904	ITS2	29631	gITS7/ITS4	16.6541°	49.3214°	Soil	7	11273	0.6209	Forest	8.5	593	NA	Czech Republic	NA
	b8f0b52dd6f8d36d54bdf8f734a52a2d	ITS2	8024	fITS7/ITS4	-1.3200°	51.7700°	Rhizosphere soil	52	20172	2.5778	Forest	9.9	707	6.3	UK	*Fraxinus excelsior*
	1599a6077fe08c03c8852c2b5037faa5	ITS2	31471	fITS7/ITS4	-1.3300°	51.7800°	Soil	62	12387	5.0052	Forest	10	698	6.2	UK	*Acer pseudoplatanus*
	abf78b869c16b36ec973096c845ea7a9	ITS2	10149	fITS7/ITS4	-1.3300°	51.7800°	Rhizosphere soil	25	19089	1.3096	Forest	10	698	5.9	UK	*Acer pseudoplatanus*
	050642e91f2b94c6a0ba6701f2233c6d	ITS2	20802	fITS7/ITS4	9.45795°	48.4013°	Soil	18	64498	0.2790	Grassland	7.8	903	5.19	Germany	NA
	39ec84a6c40ce4ed1729e50e0f16d5ea	ITS2	13560	ITS9MUNngs/ITS4ngsUni	23.5899°	58.9349°	Soil	2	4073	0.4910	Woodland	6.5	604	NA	Estonia	NA
	6bf32f8f10881becd7a8a312a6e535de	ITS2	8024	fITS7/ITS4	-1.3200°	51.7700°	Rhizosphere soil	52	20172	2.5778	Forest	9.9	707	6.3	UK	*Fraxinus excelsior*
	73c0f79ba3abf2af53d348ae51cccc78	ITS2	19751	fITS7/ITS4	10.4329°	50.9981°	Soil	7	28012	0.2498	Grassland	8.7	713	7.255	Germany	NA
	3d73800dc4c65b66ebc438ac1078b533	ITS1	1583	ITS1F/ITS4	149.5980°	-30.2007°	Soil	13	241414	0.0538	Cropland	19.3	600	7.5	Australia	NA
	908c5761a9164af02b08ef3d83349d08	ITS1	2627	ITS1F/ITS4	149.5970°	-30.2017°	Soil	6	107526	0.0558	Cropland	19.3	600	7.6	Australia	NA
Amaurascopsis perforata	b512deed3bd31f9b201c5a2c2d990206	ITS1	1583	ITS1F/ITS4	149.5980°	-30.2007°	Soil	13	241414	0.0538	Cropland	19.3	600	7.5	Australia	NA
	f640f18c3f6b36657dd41f5a0a29a184	ITS1	1906	ITS1F/ITS4	149.5970°	-30.2005°	Soil	112	260362	0.4301	Cropland	19.3	600	7.6	Australia	NA
	c7c55ca82c572aee52fb09878c6b6fff	ITS1	5523	ITS1F/ITS4	149.5970°	-30.2007	Soil	9	156916	0.0573	Cropland	19.3	600	7.6	Australia	NA
	7e9137318c201f5f9a41c2916be60068	ITS1	7440	ITS1F/ITS4	149.5970°	-30.2012°	Soil	29	246980	0.1174	Cropland	19.3	600	7.6	Australia	NA
	b7e54bc230a3d6c46a364c05c01e585c	ITS1	6165	ITS1F/ITS4	149.5970°	-30.2014°	Soil	23	317254	0.0724	Cropland	19.3	600	7.8	Australia	NA
	149a10fb0e7db6cf7d49bcdb84397b62	ITS1	26271	ITS1F/ITS4	149.5970°	-30.2007°	Soil	6	107526	0.0558	Cropland	19.3	600	7.6	Australia	NA
	22d69106778c53e0e8946c073ec2ea0e	ITS1	1906	ITS1F/ITS4	149.5970°	-30.2005°	Soil	112	260362	0.4301	Cropland	19.3	600	7.6	Australia	NA
	a29f598a9bd2986b15399f72c4c06874	ITS1	5523	ITS1F/ITS4	149.5970°	-30.2007°	Soil	9	156916	0.0573	Cropland	19.3	600	7.6	Australia	NA
	b3ba4ecea6a771b3899230555c72ac28	ITS2	29359	fITS7/ITS4	139.5400°	35.8400°	Soil	16	15573	1.0274	Cropland	15.5	1452	5.8	Japan	*Dactylis glomerata*
	998d416b16b2a109da72efa879c3fbbb	ITS2	29359	fITS7/ITS4	139.5400°	35.8400°	Soil	16	15573	1.0274	Cropland	15.5	1452	5.8	Japan	*Dactylis glomerata*
	4ad3615b1b1d5fe224922ea168dbf1f2	ITS2	29359	fITS7/ITS4	139.5400°	35.8400°	Soil	16	15573	1.0274	Cropland	15.5	1452	5.8	Japan	*Dactylis glomerata*
	7310e8324907dc77bd3df72ba1c5f30a	ITS2	29359	fITS7/ITS4	139.5400°	35.8400°	Soil	16	15573	1.0274	Cropland	15.5	1452	5.8	Japan	*Dactylis glomerata*

	4abd7f60b3103c28d95e568020b6ed7c	ITS2	29359	fITS7/ITS4	139.5400°	35.8400°	Soil	16	15573	1.0274	Cropland	15.5	1452	5.8	Japan	*Dactylis glomerata*
	51d23f0ced558145a98253a6cda44042	ITS2	29359	fITS7/ITS4	139.5400°	35.8400°	Soil	16	15573	1.0274	Cropland	15.5	1452	5.8	Japan	*Dactylis glomerata*
Apinisia racovitzae	88f8a9eb6f800f1bdb954224d03e520d	ITS2	266	ITS1F/ITS4	140.6960°	-34.0597°	Soil	98	1816901	0.0539	Shrubland	17.6	246	9	Australia	NA
	4d4068d48942e41219d09e788c4abbba	ITS2	266	ITS1F/ITS4	140.6960°	-34.0597°	Soil	98	1816901	0.0539	Shrubland	17.6	246	9	Australia	NA
	72822cd7b8841ae8c528d23a9607bc50	ITS2	266	ITS1F/ITS4	140.6960°	-34.0597°	Soil	98	1816901	0.0539	Shrubland	17.6	246	9	Australia	NA
	911d4b750e1dd09b02698e7fd09b8494	ITS2	266	ITS1F/ITS4	140.6960°	-34.0597°	Soil	98	1816901	0.0539	Shrubland	17.6	246	9	Australia	NA
	e1839424b9a0697f307ab8ad791b0172	ITS2	266	ITS1F/ITS4	140.6960°	-34.0597°	Soil	98	1816901	0.0539	Shrubland	17.6	246	9	Australia	NA
Arachnotheca glomerata	fd0cc1200ebba95d06e995052925fcc5	ITS1	12918	ITS1F/ITS4	-53.9319°	-25.8345°	Root	5	56369	0.0887	Cropland	20.9	2066	6.52	Argentina	*Ilex paraguariensis*
	183957978e865bf62e4e7ad84b22112b	ITS1	2259	ITS1F/ITS2	114.0870°	31.8661°	Soil	285	70954	4.0166	Forest	15.3	1016	4.18	China	*Quercus acutissima*
	abe274ec2bb1d9753f61e5a713382027	ITS1	23556	ITS5/ITS2	117.0100°	27.6600°	Soil	2	19664	0.1017	Forest	18.5	1737	4.53	China	*Phyllostachys pubescens*
	399ada34b884e9ae275a4e0f9c1e28da	ITS1	21395	ITS1F/ITS4	-54.1797°	-25.8669°	Root	4	31695	0.1262	Cropland	20.9	1992	6	Argentina	*Ilex paraguariensis*
	f98624a4a319927338c8734e74494b6e	ITS1	322	ITS1F/ITS2	110.4940°	31.3225°	Soil	4	40932	0.0977	Forest	10.1	1343	7.4	China	*Pinus tabuliformis*
	d9183653c8bf198a4d719e000b9bfc8a	ITS1	2259	ITS1F/ITS2	114.0870°	31.8661°	Soil	285	70954	4.0166	Forest	15.3	1016	4.18	China	*Pinus tabuliformis*
	ed2e9afeee0d682ee8592b31ac20d147	ITS1	31509	ITS1F/ITS2	119.4370°	30.3315°	Soil	471	43429	10.8452	Forest	14.5	1919	4.59	China	*Acer henry*
	e9a22612a48a86146d593d45eb6016c3	ITS1	26962	ITS1F/ITS2	114.0870°	31.8661°	Soil	6	64425	0.0931	Forest	15.3	1016	4.18	China	*Quercus acutissima*
	a76c3d4a76d7ecb10841f5f628ebd1e2	ITS1	16287	ITS1F/ITS2	147.3500°	-9.4447°	Soil	1	39759	0.0251	Forest	25.8	1969	4.46	Papuna New Guinea	*Castanopsis acuminatissima*
	d2835fa37e36fcc11abc77c13fb6fba1	ITS1	7638	ITS1F/ITS2	-95.0800°	18.5800°	Soil	4	47885	0.0835	Forest	23.7	31.55	NA	Mexico	*Astrocaryum mexicanum*
	c05a88469ce82b722db9e7d21496964c	ITS2	14553	gITS7ngs/ITS4ngsUni	101.0200°	24.5300°	Top soil	208	268230	0.7754	Forest	12.9	1557	NA	China	NA
	3306decc986eae2330d581ba38d1a4be	ITS2	4916	gITS7ngs/ITS4ngsUni	101.5700°	21.6100°	Top soil	67	978478	0.0684	Forest	21.6	1418	NA	China	NA
	d6f3fb942590df6d2d8cd6cf6122cf59	ITS2	1121	gITS7ngs/ITS4ngsUni	101.0200°	24.5300°	Top soil	1	1121182	0.0008	Forest	12.9	1557	NA	China	NA
	8cdc9982113fe617b4d61e2aa367f4a8	ITS2	2163	gITS7ngs/ITS4ngsUni	101.5700°	21.6100°	Top soil	1245	1360471	0.9151	Forest	21.6	1418	NA	China	NA
	260703140f71bee9a845306e067b7830	ITS2	22762	gITS7ngs/ITS4ngsUni	101.5700°	21.6100°	Top soil	32	19349	1.6538	Forest	21.6	1418	NA	China	NA
	316723638a37c2373ad76cbcc203a2db	ITS2	4611	gITS7ngs/ITS4ngsUni	101.5700°	21.6100°	Top soil	69	765894	0.0900	Forest	21.6	1418	NA	China	NA
	68642a10c8d66aee940cd8ce3fa2a4ee	ITS2	12361	fITS7/ITS4	-72.2079°	42.5084°	Soil	83	157622	0.5265	Forest	9.1	1280	5.2	USA	*Acer saccharum*
	7f3a45f20ba9d7aa0c7d643e34783b35	ITS2	4832	fITS9/ITS4	-89.3500°	43.1333°	Soil	12	137865	0.0870	Grassland	8.7	775	6.35	USA	*Panicum virgatum*
	cc3815a90fbc036d5563be38936da11e	ITS2	18710	gITS7ngs/ITS4ngsUni	101.5740°	21.6120°	Top soil	18	29964	0.6007	Forest	21.6	1418	NA	China	NA
	dff2bb036a25a5094ddef39f7d74f64d|	ITS2	4512	gITS7ngs/ITS4ngsUni	101.5700°	21.6100°	Top soil	1892	552679	3.4233	Forest	21.6	1418	NA	China	NA
Chrysosporium carmichaelii	623de410f842c2b393a77a6e792b8f1f|	ITS1	32793	ITS5/ITS2	107.9260°	34.5617°	Soil	3	34480	0.0870	Forest	11.7	798	NA	China	*Robinia pseudoacacia*
	579b4b33e5d353dce86dad856bdabab6	ITS1	18451	ITS5/ITS2	116.4800°	39.8800°	Air	1	49838	0.0200	Urban	12.6	609	NA	China	NA
	9230c229f798d99988799c017d01c0cf	ITS2	21478	ITS3/ITS4	117.7190°	36.9467°	Soil	1	108645	0.0092	Cropland	13.6	624	NA	China	*Triticum aestivum*
	e0b81c3305edab66c9a96b110f2475b0	ITS2	4269	ITS86F/ITS4	-3.7251°	40.4460°	Air	1	145405	0.0068	Urban	14.8	513	NA	Spain	NA
	ce596f2aeae6daf4bd9b1f86dd0a2c15	ITS2	7507	ITS3_KYO2/ITS4	-99.1761°	19.3264°	Air	2	97838	0.0204	Urban	16.4	927	NA	Mexico	NA
	9a20c196e3bce863ba7722ecd7b945d6	ITS2	19143	ITS3ngs1 to 5 + ITS3ngs10/ITS4ngs	121.4000°	34.0000°	Water	3	14534	0.2064	Aquatic	NA	NA	NA	China	NA
	346a4c389d997812a4cd7771c7152c6e	ITS2	21452	ITS3/ITS4	117.7050°	37.0293°	Soil	4	70545	0.0567	Cropland	13.5	638	NA	China	NA
	9def6bc05d6636b59461ce0e70b657f7	ITS2	37654	ITS3ngs1 to 5 + ITS3ngs10/ITS4ngs	122.3300°	36.0000°	Water	1	18230	0.0548	Aquatic	NA	NA	NA	China	NA
	b87ce203bdee7a520992103cc96b1ca2	ITS2	19143	ITS3ngs1 to 5 + ITS3ngs10/ITS4ngs	121.4000°	34.0000°	Water	3	14534	0.2064	Aquatic	NA	NA	NA	China	NA

Chrysosporium chiropterum	e1980d9fafeb0cc21b6a5a18d1925279	ITS1	26731	ITS1F/ITS4	28.4889°	-25.9278°	Soil	3	50047	0.0599	Grassland	16.1	706	6.5	South Africa	*Acacia dealbata*
	02c4229e65aadc67efd43bbe6725c164	ITS1	17260	ITS3_KYO2/ITS4	-2.77837°	42.5060°	Soil	23	83881	0.2741	Cropland	12.9	735	8.2	Spain	*Vitis vinifera*
	90181ed69e2c06f573e77f90cd70bfde	ITS1	11494	ITS3_KYO2/ITS4	-2.8528°	42.5928°	Root	21	123909	0.1694	Cropland	13	598	8.2	Spain	*Vitis vinifera*
	2d4f3dde60e993bfb72e2d1453ffc8e7	ITS1	25873	ITS5/ITS2	108.4680°	36.0740°	Soil	1	51985	0.0192	Cropland	8.8	625	8.33	China	*Zea mays*
	afc2ab4bba5a7fea8a294f9ba39c104a	ITS2	24407	fITS7/ITS4	9.3984°	48.3699°	Soil	3	47464	0.0632	Grassland	7.9	792	6.15	Germany	NA
	7087f1c8f84ae8efca408fe0a0cd2d89	ITS2	15173	ITS3/ITS4	128.4630°	38.0321°	Rhizosphere soil	4	194956	0.0205	Forest	8.1	1382	5.7	South Korea	*Carpinus cordata*
	7087f1c8f84ae8efca408fe0a0cd2d89	ITS2	15173	ITS3/ITS4	128.4630°	38.0321°	Rhizosphere soil	4	194956	0.0205	Forest	8.1	1382	5.7	South Korea	*Carpinus cordata*
	afc2ab4bba5a7fea8a294f9ba39c104a	ITS2	24407	fITS7/ITS4	9.3984°	48.3699°	Soil	3	47464	0.0632	Grassland	7.9	792	6.15	Germany	NA
Chrysosporium lobatum	c4ab8f17decdcbe21ee81092e4561428	ITS1	9875	ITS5/ITS2	107.9260°	34.5567°	Soil	1	21598	0.0463	Forest	12	785	NA	China	*Robinia pseudoacacia*
	21505d4a40c013eb54a57750fc14c896	ITS1	22495	ITS1F/ITS2	116.8260°	28.2289°	Soil	19	56117	0.3385	Cropland	19.2	1748	5.53	China	*Oryza sativa*
	c064f9081f884a16cb367663bb778651	ITS1	18934	ITS5/ITS2	117.2000°	34.3400°	Soil	103	82235	1.2525	Cropland	14.7	842	7.79	China	*Zea mays*
	98a48944c14e94e97e55407525653bc0	ITS1	25066	ITS5/ITS2	116.4800°	39.8800°	Air	2	49992	0.0400	Urban	12.6	609	NA	China	NA
	6d9c45a27f39b09557727503aff1aad5	ITS1	18934	ITS5/ITS2	117.2200°	34.3400°	Soil	103	82235	1.2525	Cropland	14.7	842	7.79	China	*Zea mays*
	6c11b5978d2a3869c90b0c0619218010	ITS1	18934	ITS5/ITS2	117.2200°	34.3400°	Soil	103	82235	1.2525	Cropland	14.7	842	7.79	China	*Zea mays*
	40b3daa50de24d9bb0e7024d78614dd5	ITS1	22495	ITS1F/ITS2	116.8260°	28.2289°	Soil	19	56117	0.3385	Cropland	19.2	1748	5.53	China	*Oryza sativa*
	258d0e5184ea34997c5bb22414dc0d09	ITS1	22495	ITS1F/ITS2	116.8260°	28.2289°	Soil	19	56117	0.3385	Cropland	19.2	1748	5.53	China	*Oryza sativa*
	0ee0e5bb36477433a9273018b053e6ed	ITS1	22495	ITS1F/ITS2	116.8260°	28.2289°	Soil	19	56117	0.3385	Cropland	19.2	1748	5.53	China	*Oryza sativa*
	803926142f0412c70ffbb625acf90442	ITS1	24248	ITS5/ITS2	106.2200°	38.3100°	Soil	13	68262	0.1904	Cropland	10.5	201	8.37	China	*Zea mays*
	9e51dc2a8d2dc9b9b514b1b93a7dbaa3	ITS2	22592	ITS3ngs1 to 5 + ITS3ngs10/ITS4ngs	120.7600°	38.3459°	Water	128	20989	6.0984	Aquatic	NA	NA	NA	China	NA
	6fce35a01b556394b40dbd2fe88f2e11	ITS2	21465	ITS3/ITS4	113.0900°	28.2000°	Soil	1	15126	0.0661	Cropland	18.4	1473	6.55	China	*Glycine max*
	63ad5c9d4d09b84ccb50a59149397b47	ITS2	22592	ITS3ngs1 to 5 + ITS3ngs10/ITS4ngs	120.7600°	38.3459°	Water	128	20989	6.0984	Aquatic	NA	NA	NA	China	NA
	ecc0c76261ee19696608f181e3431227	ITS2	22592	ITS3ngs1 to 5 + ITS3ngs10/ITS4ngs	120.7600°	38.3459°	Water	128	20989	6.0984	Aquatic	NA	NA	NA	China	NA
	e9d6fb53070a18dc55e7a39fd4ddfb4a	ITS2	9936	ITS3/ITS4	-83.9600°	35.8400°	Rhizosphere soil	3	132473	0.0226	Forest	14.9	1192	NA	China	*Glycine max*
	2c143f7424e2b36fbb351271fbe08c06	ITS2	4606	ITS3/ITS4	-83.9600°	35.8400°	Rhizosphere soil	4	240124	0.0166	Forest	14.9	1192	NA	USA	*Populus deltoides*
	4cab851e82c0d2b1e3f87280819a10d5	ITS2	22592	ITS3ngs1 to 5 + ITS3ngs10/ITS4ngs	120.7600°	38.3459°	Water	128	20989	6.0984	Aquatic	NA	NA	NA	China	NA
	d8255312c75e23089e99c9cf54b68e57	ITS2	22592	ITS3ngs1 to 5 + ITS3ngs10/ITS4ngs	120.7600°	38.3459°	Water	128	20989	6.0984	Aquatic	NA	NA	NA	China	NA
	14c615c7de320ecd8d2fc3a333edb908	ITS2	29732	ITS3ngs1 to 5 + ITS3ngs10/ITS4ngs	119.2040°	38.3190°	Water	1	110017	0.0090	Aquatic	NA	NA	NA	China	NA
	0228cc1f6afd028d3024891a776c5164|	ITS2	22592	ITS3ngs1 to 5 + ITS3ngs10/ITS4ngs	120.7600°	38.3459°	Water	128	20989	6.0984	Aquatic	NA	NA	NA	China	NA
Chrysosporium pallidum	546e8b62b169a16935c46b3c660456a0	ITS2	34938	ITS3/ITS4	114.8560°	37.8031°	Rhizosphere soil	1	50548	0.0197	Cropland	14.5	521	NA	China	NA
Chrysosporium sulfureum	6a4c0176bffb4b9b4cf6aa004fc2bf51	ITS2	23040	ITS3/ITS4	10.4656°	51.1129°	Air	2	24090	0.0830	Forest	8.7	554	NA	Germany	NA
	1d5f60a0df5cad7bc1561b0e2216efdd	ITS2	23040	ITS3/ITS4	10.4656°	51.1129°	Air	2	24090	0.0830	Forest	8.7	554	NA	Germany	NA
Chrysosporium undulatum	b1676d95bf4d8bef02e8d020daf54120	ITS1	47114	ITS9MUNngs/ITS4ngsUni	22.2143°	58.1157°	Soil	6	2390	2.5104	Forest	7	590	NA	Estonia	NA
	de1309038b56fb89abee5f547d87b0a2	ITS1	22563	ITS9MUNngs/ITS4ngsUni	14.8295°	49.7167°	Soil	2	4657	0.4294	Forest	8.5	549	NA	Czech Republic	NA
	0897ada14dc18abdd24ebdc3529fb391	ITS1	18470	ITS9MUNngs/ITS4ngsUni	12.9883°	49.5452°	Soil	5	10479	0.4771	Forest	8.4	730	NA	Czech Republic	NA
	092623792d0f2c4f74010f6b1f0b7f36	ITS1	33900	ITS9MUNngs/ITS4ngsUni	15.0201°	50.2907°	Soil	12	9549	1.2566	Forest	8.9	634	NA	Czech Republic	NA
	15023ddfdb03b16e622ff5e1fc1f8d23	ITS1	26729	ITS9MUNngs/ITS4ngsUni	14.1076°	49.4373°	Soil	6	4997	1.2007	Forest	8.6	546	NA	Czech Republic	NA

	6c41ac7d66368aa24d4874ed0e0ce861	ITS1	29460	ITS9MUNngs/ITS4ngsUni	26.8737°	57.9812°	Soil	12	6605	1.8168	Urban	5.4	621	NA	Estonia	NA
	1b9f034469e42c55b17a79e50dac792b	ITS1	26311	ITS9MUNngs/ITS4ngsUni	14.7831°	48.9399°	Soil	10	39009	0.2563	Forest	8.7	627	NA	Czech Republic	NA
	5517108db0442e11b505401ac9273cd8	ITS1	22275	ITS9MUNngs/ITS4ngsUni	24.9002°	58.9071°	Soil	1	8939	0.1118	Forest	5.7	722	NA	Estonia	NA
	7bb99f2ca2c88020ffdfcb739ba9536e	ITS1	23342	ITS9MUNngs/ITS4ngsUni	12.6092°	50.0069°	Soil	3	8615	0.3482	Forest	7.1	757	NA	Czech Republic	NA
	f2814c88e8dce322b35b32823f7a42f3	ITS1	26311	ITS9MUNngs/ITS4ngsUni	14.7831°	48.9398°	Soil	10	39009	0.2563	Forest	8.7	627	NA	Czech Republic	NA
	72c90ba34eacb80756a14a0b130c651c	ITS2	40688	gITS7/ITS4	16.6541°	49.3214°	Soil	1	5570	0.1795	Forest	8.5	593	4.51	Czech Republic	*Picea abies*
	a78576e908a8ce117ae54a908919faa8	ITS2	47114	ITS9MUNngs/ITS4ngsUni	22.2143°	58.1570°	Soil	6	2393	2.5073	Forest	7	590	NA	Czech Republic	*Picea abies*
	5e5d51e7eec6c594ff8109da8839fd2e	ITS2	35933	gITS7/ITS4	16.6488°	492.6160°	Litter	1	9353	0.1069	Forest	8.8	558	6.5	Czech Republic	*Picea abies*
	257aa333e9795ac155fb2c42d56519b3	ITS2	5981	fITS7/ITS4	147.3880°	-33.8449°	Soil	1	53832	0.0185	Forest	16.8	470	6.74	Australia	NA
	0325a48229e0434ceafe591724f16c50	ITS2	27582	gITS7/ITS4	14.7036°	-48.6660°	Deadwood	2	21583	0.0926	Forest	6.6	784	3.73	Australia	NA
	c4d64d6c836a83e38ae2fbd2d85fc2a2	ITS2	6814	ITS1F/ITS4	9.4392°	48.3959°	Rhizosphere soil	3	74470	0.0402	Grassland	7.8	903	NA	Germany	*Dactylis glomerata*
	0199e4317cb7a44e1042779b92fe8867	ITS2	9671	gITS7/ITS4	16.6452°	49.3125°	Soil	3	57454	0.0522	Forest	9.2	485	5.43	Czech Republic	*Fagus sylvatica*
	4ba6ca174787dfe83a49995fa9e4e9c3	ITS2	29544	ITS9MUNngs/ITS4ngsUni	14.5397°	49.9911°	Soil	1	18500	0.0540	Forest	8.7	559	NA	Czech Republic	NA
	698f5dc86e62d6e5322cefd0385ad20b	ITS2	11271	fITS7/ITS4	149.8880°	-34.7437°	Soil	126	70143	1.7963	Forest	13	680	5.52	Australia	NA
	a21e601967f9974c2cdc98802c268cc2	ITS2	31445	TS9MUNngs/ITS4ngsUni	16.9944°	49.9760°	Soil	9	16128	0.5580	Forest	8.4	695	NA	Czech Republic	NA
Malbranchea albolutea	46997959ae9c1e4172414dc3893c61b0	ITS1	12435	ITS1F/ITS2	82.4000°	44.9000°	Soil	794	45884	17.3045	Desert	10.8	130	8.42	China	*Haloxylon ammodendron*
	146d3e6b4e8bf8a8b284d506f7b0044f	ITS1	34411	ITS1F/ITS4	-0.5990°	39.8100°	Soil	2	8745	0.2287	Shrubland	13.3	480	7.8	Spain	*Fagaceae*
	16f07fffa4eec06a7e8a380f6ed48416	ITS1	12435	ITS1F/ITS2	82.4000°	44.9000°	Soil	794	45884	17.3045	Desert	10.8	130	8.42	China	*Haloxylon ammodendron*
	1d2c7a0fea0506e5fd175f3a3173ca6e	ITS1	13287	ITS1F/ITS2	91.1000°	43.7000°	Soil	103	31564	3.2632	Grassland	4.6	118	7.9	China	*Stipa capillata*
	25cb0da9798991f2905a4cca2539b0cf	ITS1	12435	ITS1F/ITS2	82.4000°	44.9000°	Soil	794	45884	17.3045	Desert	10.8	130	8.42	China	*Haloxylon ammodendron*
	5857c3a95b20211fda7faa5b2a2b127b	ITS1	11200	ITS1F/ITS2	107.9200°	34.5600°	Soil	9	50702	0.1775	Forest	11.5	825	NA	China	*Robinia pseudoacacia*
	6eb3731c69b517de0ed3a5be8d6b4da0	ITS1	37389	ITS1F/ITS2	84.9000°	44.1000°	Soil	1	35492	0.0281	Grassland	3.6	177	7.9	China	*Stipa capillata*
	8670583bec42d296bc0a1a4c27f87c53	ITS1	12435	ITS1F/ITS2	82.4000°	44.9000°	Soil	794	45884	17.3045	Desert	10.8	130	8.42	China	*Haloxylon ammodendron*
	a39aed4f0c8508d03e87a4e376a09e74	ITS2	34205	fITS7/ITS4	-96.6122°	39.1020°	Soil	1	20261	0.04935	Grassland	12.9	748	6.31	USA	*Juniperus virginiana*
	f350ebf031ee199def3bb8d278983d8d	ITS2	9287	fITS7/ITS4	-3.5587°	33.9326°	Soil	678	70531	9.6127	Desert	18.6	292	8.48	Morocco	NA
	8c5104a2e7211a4ecff4d04644dfc6b1	ITS2	15620	fITS7/ITS4	-110.5170°	38.0031°	Soil	35	139659	0.2506	Shrubland	13.9	120	8.88	USA	NA
	6805f6586043b848caa174d639a4721a	ITS2	24732	ITS4_Fun/5.8S_Fun	-111.5680°	35.5722°	Soil	46	21121	2.1779	Desert	12.3	284	NA	USA	NA
	30874049868f2a52ad99a5e921bef5df	ITS2	15620	fITS7/ITS4	-110.5170°	38.0031°	Soil	35	139659	0.2506	Shrubland	13.9	120	8.88	USA	NA
	fc3ad7f45edf6483339157726ac51223	ITS2	9287	fITS7/ITS4	-3.5587°	33.9326°	Soil	678	70531	9.6127	Desert	18.6	292	8.48	Morocco	NA
	41fdf6f4ed1bc9c509dbd75a47331033	ITS2	20303	fITS7/ITS4	-1.9993°	34.3097°	Soil	341	99995	3.4101	Grassland	14.9	234	8.31	Morocco	NA
	d4589bc2af525eff8db27770d95e777f|	ITS2	16669	fITS7/ITS4	-2.3729°	34.1592°	Soil	150	66491	2.2559	Desert	16.4	278	8.30	Morocco	NA
	986d2513ffd366edd7fe143ea1aca01f|	ITS2	9278	fITS7/ITS4	-1.9993°	34.3097°	Soil	275	66131	4.1584	Desert	14.9	324	8.36	Morocco	NA
	d89b9e4c2b3d6b51441990f5132ca07e	ITS2	12718	fITS7/ITS4	-112.5520°	37.1134°	Soil	406	126190	3.2173	Shrubland	12.3	265	8.44	USA	NA
Malbranchea californiensis	ec466e9e168787b69805b9c461d33f2e	ITS1	10840	ITS5/5.8S_fungi	-3.1166°	52.9072°	Soil	109	213318	0.5109	Grassland	8.2	961	3.57	UK	NA
	1fcdc37fa2dd49a1df55867c5e2a474f	ITS1	27215	ITS9MUNngs/ITS4ngsUni	13.8131°	48.9810°	Soil	93	14704	6.3248	Forest	5	1060	NA	Czech Republic	NA
	d176efcd6f205c56536090e1d37b538b	ITS1	22563	ITS9MUNngs/ITS4ngsUni	14.8295°	49.7167°	Soil	2	4657	0.4294	Forest	8.5	549	NA	Czech Republic	NA

	512a1da49a8b46883a736f2166f66ec1	ITS1	2939	ITS1F/ITS2	4.5610°	51.0720°	Root	1	83319	0.0120	Forest	10.6	814	4.21	Belgium	*Quercus robur*
	38902800add46ef952539444b2fff787	ITS1	13689	ITS5/5.8S_fungi	-3.5434°	52.3630°	Soil	4	230670	0.0173	Grassland	7.7	1963	4.73	UK	NA
	4e7eec86b15ef9c22de97c60958333fd	ITS1	27215	ITS9MUNngs/ITS4ngsUni	13.8131°	48.9810°	Soil	93	14704	6.3248	Forest	5	1060	NA	Czech Republic	NA
	4829f5243d075652da433cdf92925a28	ITS1	27215	ITS9MUNngs/ITS4ngsUni	13.8131°	48.9810°	Soil	93	14704	6.3248	Forest	5	1060	NA	Czech Republic	NA
	87b1fd3c3c9e5ff5260e18be31d3a636	ITS1	27215	ITS9MUNngs/ITS4ngsUni	13.8131°	48.9810°	Soil	93	14704	6.3248	Forest	5	1060	NA	Czech Republic	NA
	2a7b98ff57075d3468f5d49fef537ff3	ITS1	27215	ITS9MUNngs/ITS4ngsUni	13.8131°	48.9810°	Soil	93	14704	6.3248	Forest	5	1060	NA	Czech Republic	NA
	149b4d85d17769cbc1d67bb350cff3ab	ITS1	27215	ITS9MUNngs/ITS4ngsUni	13.8131°	48.9810°	Soil	93	14704	6.3248	Forest	5	1060	NA	Czech Republic	NA
	1b9d97117a12d57fd51242dba837eaec	ITS2	9651	glts7ngs/ITS4ngsUni	101.0160°	24.5330°	Top soil	26	35061	0.7415	Forest	13	1546	NA	China	NA
	93b48a19a9a56e3af058ed31a904f731	ITS2	82752	glts7ngs/ITS4ngsUni	101.0200°	24.5300°	Top soil	421	949101	0.4435	Forest	12.9	1557	NA	China	NA
	06027f3e4e0301b2fc39759745356d85	ITS2	6604	glts7ngs/ITS4ngsUni	101.0200°	24.5300°	Top soil	5379	1199523	4.4842	Forest	12.9	1557	NA	China	NA
	03b49e7c95f2eee274b8527cc0155e31	ITS2	6604	glts7ngs/ITS4ngsUni	101.0200°	24.5300°	Top soil	5379	1199523	4.4842	Forest	12.9	1557	NA	China	NA
	1f673d5651ace6c9c3f8b7a523af0e33	ITS2	106	glts7ngs/ITS4ngsUni	101.0160°	24.5330°	Top soil	50	115411	0.4332	Forest	13	1546	NA	China	NA
	57afd1bf8841015f97f9728088416b99	ITS2	15225	glts7ngs/ITS4ngsUni	101.0200°	24.5300°	Top soil	109	700669	0.1555	Forest	12.9	1557	NA	China	NA
	5f891f54c11d1ca5360a24ee84dde92d	ITS2	2556	glts7ngs/ITS4ngsUni	101.0200°	24.5300°	Top soil	4	568348	0.0070	Forest	12.9	1557	NA	China	NA
	88cff43503f9c8a0b9be5b52b31c177a	ITS2	1199	glts7ngs/ITS4ngsUni	101.0200°	24.5300°	Top soil	503	843445	0.5963	Forest	12.9	1557	NA	China	NA
	c901854d2ead1a26753299e4784d32fa	ITS2	7217	glts7ngs/ITS4ngsUni	101.0200°	24.5300°	Top soil	218	1006058	0.2166	Forest	12.9	1557	NA	China	NA
	d2dda8d887de629107c6b97ad67e1db8	ITS2	6602	glts7ngs/ITS4ngsUni	101.0200°	24.5300°	Top soil	5348	1263089	4.2340	Forest	12.9	1557	NA	China	NA
Malbranchea chinensis	2c3d4f0c6bb747033d495d64002407ef	ITS2	12973	fITS9/ITS4	-70.8394°	-27.4675°	Soil	751	206768	3.6320	Desert	16.8	26	9.24	Chile	*Cistanthe longiscapa*
	4eca68b22046729655cd343214ffd9e3	ITS2	17587	fITS9/ITS4	-70.8394°	-27.4676°	Root and Rhizosphere soil	785	193894	4.0486	Desert	16.8	26	9.24	Chile	*Cistanthe longiscapa*
	56d65a3412420af5e562357db844af11	ITS2	3493	ITS3ngs1 to 5 + ITS3ngs10/ITS4ngs	123.0000°	35.0000°	Water	2	14142	0.1414	Marine	NA	NA	NA	China	NA
	5711d54dc1b835e020a0b8c8b30f134b	ITS2	1282	ITS86F/ITS4	-2.9861°	37.0064°	Rhizosphere soil	12	149311	0.0803	Shrubland	13.2	446	7.95	Spain	*Thymus zygis*
	66b7e126ab05193ddd26a24ea68eaa52	ITS2	9292	ITS86F/ITS4	-1.1627°	34.0334°	Rhizosphere soil	1	137515	0.0072	Shrubland	18.5	299	8.46	Spain	*Thymalea hirsuta*
	7eff344b359457a4dff393af5d7d95fd	ITS2	17587	FLTS9/ITS4	-70.8394°	-27.4676°	Root and Rhizosphere soil	785	193894	4.0486	Desert	16.8	26	9.24	Chile	*Cistanthe longiscapa*
	8181f4f83d841895fb6a7b14fd03cf65	ITS2	12973	FLTS9/ITS4	-70.8394°	-27.4675°	Soil	751	206768	3.6320	Desert	16.8	26	9.24	Chile	*Cistanthe longiscapa*
	8f0f64ace6ef0240af70a206eb20247f	ITS2	34938	ITS3ngs1 to 5 + ITS3ngs10/ITS4ngs	123.5000°	35.0000°	Water	2	14142	0.1414	Aquatic	NA	NA	NA	China	NA
	9f9c306686a43a6ea38671dfd510547e	ITS2	589	Clts9/ITS4	107.5000°	35.6700°	Soil	2215	213525	10.3734	Desert	NA	NA	NA	China	NA
	ee3be12b37d7cc6f7e0fecab6b4bbd5a	ITS2	17587	Clts9/ITS4	107.5000°	35.6700°	Root and Rhizosphere soil	785	193894	4.0486	Desert	NA	NA	NA	China	NA
Malbranchea compacta	3441eb3dbd4bff53efe53a4579fc6a20	ITS1	10297	ITS5/ITS4	-111.5680°	35.5722°	Soil	21	4936	4.2544	Desert	8.5	284	7.9	China	*Potentilla bifurca*
	e659c8ae27cf0cd0de14465cf8f76613	ITS1	6561	ITS5/ITS4	-111.5680°	35.5722°	Soil	22	6896	3.1902	Desert	8.5	284	7.9	China	*Stipa grandis*
	b5b1a4d1d6152cc1b56b0d2bb1fe3a98	ITS1	9123	ITS5/ITS4	-111.5680°	35.5722°	Soil	7	4443	1.5755	Desert	8.5	284	7.9	China	*Stipa grandis*
	160729c8b1e82065696f07dbe9bd6daa	ITS1	30540	ITS5/ITS4	-111.5680°	35.5722°	Soil	2	5845	0.3421	Desert	8.5	284	7.9	China	*Bouteloua eriopoda*
	3653cf5ee48e5bad251acd299a91712b	ITS1	20253	ITS5/ITS4	-111.5680°	35.5722°	Soil	5	7741	0.6459	Desert	8.5	284	7.9	China	*Bouteloua eriopoda*
	4e7433e58d6d971bcfc47acfa6929efd	ITS1	10297	ITS5/ITS4	-111.5680°	35.5722°	Soil	21	4936	4.2544	Desert	8.5	284	7.9	China	*Bouteloua eriopoda*
	936d44c7a9398cd2b665032a8374e5f8	ITS1	10297	ITS5/ITS4	-111.5680°	35.5722°	Soil	21	4936	4.2544	Desert	8.5	284	7.9	China	*Bouteloua eriopoda*
	9518f772616f9fb7d686094bd0420d85	ITS1	6561	ITS5/ITS4	-111.5680°	35.5722°	Soil	22	6896	3.1902	Desert	8.5	284	7.9	China	*Bouteloua eriopoda*

	b4bf4d87fc6b59967e36d9c32b25dbaf	ITS1	10297	ITS5/ITS4	-111.5680°	35.5722°	Soil	21	4936	4.2544	Desert	8.5	284	7.9	China	*Bouteloua eriopoda*
	bf10747fd122c6df4de25f2932d86a68	ITS1	20253	ITS5/ITS4	-111.5680°	35.5722°	Soil	5	7741	0.6459	Desert	8.5	284	7.9	China	*Bouteloua eriopoda*
	807de4363a2bebd5bfabd3e31975c663	ITS2	18036	fITS7/ITS4	-112.0220°	37.5073°	Soil	16	99759	0.1603	Shrubland	11.6	208	8.27	Australia	NA
	d59acf27406f4525d1f58425507d2d66	ITS2	38925	ITS4_Fun/5.8S_Fun	-111.5680°	35.5722°	Soil	12	23196	0.5173	Desert	12.3	284	NA	USA	NA
	f6bb539ca3a49dc8fed8176d08e6dbeb	ITS2	8521	ITS1F/ITS4	148.7990°	-35.4214°	Soil	1	247458	0.0040	Forest	9.2	12.21	5.7	Australia	NA
	0b29b8b0a58e5f449d04d2e1095151b8	ITS2	11368	ITS1F/ITS4	116.1210°	-32.5668°	Soil	1	85183	0.0117	Forest	16.6	111	5.7	Australia	NA
	224e02ea3652ecdd154ed6b69e47fad2	ITS2	38925	ITS4_Fun/5.8S_Fun	-111.5680°	35.5722°	Soil	12	23196	0.5173	Desert	12.3	284	NA	USA	NA
	24c9b467a52bc6f686c125282369efb3	ITS2	38925	ITS4_Fun/5.8S_Fun	-111.5680°	35.5722°	Soil	12	23196	0.5173	Desert	12.3	284	NA	USA	NA
	891f4e1ac4b2d6817e2413b6378e9af3	ITS2	7687	ITS1F/ITS4	132.2240°	-30.9014°	Soil	1	99489	0.0100	Shrubland	18	251	9.4	Australia	NA
	9a366b4cbcbdb40340dae6d493b76461	ITS2	1161	ITS1F/ITS4	132.1980°	-30.8871°	Soil	1	89984	0.0111	Desert	18.2	229	9	Australia	NA
	bf043a995eda08cc3ce7257ddab9b2cd	ITS2	18036	fITS7/ITS4	-112.0220°	37.5073°	Soil	16	99759	0.1603	Shrubland	11.6	208	8.27	USA	NA
	ca068179681c4fbf6448a9392101a2b9	ITS2	11205	ITS1F/ITS4	-145.0290°	-36.6732°	Soil	25	255464	0.0978	Woodland	15.1	530	5.5	Chile	*Cistanthe longiscapa*
Malbranchea ostraviensis	da95aec3cba7c9d7defe2155f2708d1b	ITS1	266	ITS1F/ITS4	140.6960°	-34.0597°	Soil	744	1816901	0.4094	Shrubland	17.6	246	9	Australia	NA
	e2331d285395d127637a255e5313362d	ITS1	30533	ITS1F/ITS4	-0.21350°	38.8145°	Soil	6	16411	0.3656	Shrubland	14.5	700	7	Spain	*Fagaceae*
	fab7073bcf7bcb14c24b05f37dbcc8cb	ITS1	38755	ITS5/ITS2	107.5000°	35.6700°	Soil	193	29661	6.5068	Grassland	10.9	403	NA	China	*Artemisia vestita*
	8efc0fe7a2ffe243803e6250e59e37c6	ITS1	7727	ITS1F/ITS4	138.1300°	-35.6407°	Soil	37	107794	0.3432	Woodland	15.3	673	8.3	Australia	NA
	88a7a5c5b7c4748852045d00b1f4b704	ITS1	24340	ITS1F/ITS2	115.4290°	39.9613°	Soil	34	76690	0.4433	Forest	5.8	549	6.81	China	*Acer truncatum*
	f7de87dcf3f04a2947c052c37a90e8b2	ITS1	704	ITS5/ITS2	88.0600°	44.4100°	Soil	61	65664	0.9289	Desert	9.5	150	7.81	China	*Seriphidium transiliense*
	65c316ed491497d38738aee4e63d0122	ITS1	266	ITS1F/ITS4	140.6960°	-34.0597°	Soil	744	1816901	0.4094	Shrubland	17.6	246	9	Australia	NA
	c5b135116f3e42662adfbe278d6bb704	ITS1	266	ITS1F/ITS4	140.6960°	-34.0597°	Soil	744	1816901	0.4094	Shrubland	17.6	246	9	Australia	NA
	49de2234c95ff76b2686a566cbacb7ac	ITS1	2755	ITS1F/ITS4	126.9830°	-31.8633°	Soil	749	154275	4.8549	Shrubland	17.9	272	8.6	Australia	NA
	e49f9e99a02467d8add8a8dd5d39de0d	ITS1	38755	ITS5/ITS2	107.5000°	35.6700°	Soil	193	29661	6.5068	Grassland	10.9	403	NA	China	*Artemisia vestita*
	756bbeb0cc007f63c2d532ed2e0b50ff	ITS2	3771	ITS3ngs1 to 5 + ITS3ngs10/ITS4ngs	-5.6785°	-15.9489°	Soil	4	31158	0.1283	Woodland	18.8	892	5.75	Saint Helena, UK	*Commidendrum robustum*
	6a9cdac94c3c574f6eed3f8b710225fe	ITS2	6651	ITS86F/ITS4	-3.3548°	36.9241°	Soil	124	168805	0.7345	Shrubland	13.7	498	7.24	Spain	*Thymus zygis*
	4d2b1c5da794d1f3dedd25641141889a	ITS2	1019	fITS7/ITS4	-64.9345°	-23.9188°	Soil	478	235226	2.0320	Forest	19.8	739	6.76	Argentina	*Calycophy* sp.
	3629c91686004069fe1bb36c2eec7da8	ITS2	1019	fITS7/ITS4	-64.9345°	-23.9188°	Soil	478	235226	2.0320	Forest	19.8	739	6.76	Argentina	*Calycophy* sp.
	29ecb1dc973d7d84d0c4c4d5abf8d4f0	ITS2	2065	fITS7/ITS4	-64.8503°	-23.7571°	Soil	249	139337	1.7870	Forest	20.2	752	6.85	Argentina	*Calycophyllum multiflorum*
	0a4574962adb57149453ccd4018f5d94	ITS2	3773	ITS3/ITS4	-5.6785°	-15.9489°	Soil	2	287758	0.0069	Forest	18.8	892	5.75	Saint Helena, UK	NA
	0553eeb1a85066ed6bde7c96c5732c1f|	ITS2	6186	ITS1F/ITS4	9.4890°	39.8060°	Soil	1	207333	0.0048	Cropland	15.5	632	8.1	Ethiopia	NA
	d0961116d67b4b46b52ff86b091db60a	ITS2	1019	fITS7/ITS4	-64.9345°	-23.9188°	Soil	478	235226	2.0320	Forest	19.8	739	6.76	Argentina	*Calycophy sp.*
	1895b6c87131814ff4bcad198008cadb	ITS2	6186	ITS1F/ITS4	9.4890°	39.8060°	Soil	1	207333	0.0048	Cropland	15.5	632	8.1	Ethiopia	NA
	fce2849c9bd9bc6bb1883c0cea5e1f5f	ITS2	7529	ITS1F/ITS4	143.9730°	-39.6509°	Soil	104	304789	0.3412	Woodland	13.6	898	6.3	Australia	NA
Malbranchea reticulata	5d3e2f00b11b8a7d548a666aab7f94ae	ITS1	30015	ITS5/ITS4	-2.3773°	42.5406°	Rhizosphere soil	9	98405	0.0914	Cropland	13	626	8.2	Spain	*Vitis vinifera*
	ae0dae2a04eb077edae2edca26f4e64	ITS1	7295	ITS5/ITS4	-2.77837°	42.5060°	Root	36	137225	0.2623	Cropland	12.9	735	8.2	Spain	*Vitis vinifera*
	fc51339587df9c6b0c0e96cffb3f7134	ITS1	25274	ITS5/ITS4	-2.7783°	42.5060°	Soil	1	99656	0.0100	Cropland	12.9	735	8.2	Spain	*Vitis vinifera*
	36582ea8149842112f925cf5bd4fb7bf	ITS1	29875	ITS5/ITS4	-0.6056°	39.8002°	Soil	2	13115	0.1524	Shrubland	13.7	451	8.2	Spain	*Fagaceae*

	2de5f5c7fe5cdd8f6709262f7259c64e	ITS1	29212	ITS5/ITS4	106.3910°	36.2850°	Soil	220	82126	2.6788	Grassland	6.2	472	8.4	China	*Potentilla bifurca*
	fea93efbc361fd6342f8ab629b340788	ITS1	30015	ITS5/ITS4	-2.7738°	42.5406°	Rhizosphere soil	9	98405	0.0914	Rhizosphere soil	13	626	8.2	Spain	*Vitis vinifera*
	d20fec209db7ea37996ed7797d6e30e8	ITS1	7295	ITS5/ITS4	-2.7783°	42.5060°	Root	36	137225	0.2623	Cropland	12.9	735	8.2	Spain	*Vitis vinifera*
	b97a6a1d8852297bcc309fd116cbdbd1	ITS1	27425	ITS5/ITS4	-2.8695°	42.5860°	Rhizosphere soil	85	116060	0.7323	Crolpand	13	601	8,1	Spain	*Vitis vinifera*
	01bdacc124d1ede25d8196d035066359	ITS1	30015	ITS5/ITS4	-2.7738°	42.5406°	Soil	9	98405	0.0914	Cropland	13	626	8,2	Spain	*Vitis vinifera*
	68e518b58c32fdd9979a00a069cec2a0	ITS1	23595	ITS5/ITS4	-2.7738°	42.5406°	Soil	9	98405	0.0914	Cropland	13	626	8,2	Spain	*Vitis vinifera*
Malbranchea sinuata	14fed96baab05ea2c0fcd2b98e90504e	ITS2	2332	fITS7/ITS4	-0.5990°	39.8100°	Soil	8	59966	0.1334	Shrubland	13.7	498	7.24	Spain	NA
	359dd2964540ffdb3b3b3f32a12cb16f|	ITS2	2332	fITS7/ITS4	-0.5990°	39.8100°	Soil	8	59966	0.1334	Shrubland	13.7	498	7.24	Spain	NA
	48b0121f63efd52b66d2304b7cb45a68	ITS2	10269	ITS86F/ITS4	-0.5990°	39.8100°	Rhizosphere soil	1613	152304	10.5906	Shrubland	13.7	498	7.24	Spain	NA
	4f177c8e38014e0cd15d605d47bc791c	ITS2	17695	ITS86F/ITS4	-0.5990°	39.8100°	Rhizosphere soil	30	46082	0.6510	Shrubland	13.7	498	7.24	Spain	NA
	5853d823431a49fbd9f7b1e14f831aa9	ITS2	15421	fITS7/ITS4	-0.5990°	39.8100°	Soil	1	83418	0.0119	Shrubland	13.7	498	7.24	Spain	NA
	97c18eeb8268a6e478d65917fe22c99d	ITS2	10269	ITS86F/ITS4	-0.5990°	39.8100°	Rhizosphere soil	1613	152304	10.5906	Shrubland	13.7	498	7.24	Spain	NA
	cb7fbfa4ba2ef68f259de7695dd758c5	ITS2	4008	ITS86F/ITS4	-0.5990°	39.8100°	Rhizosphere soil	13	175789	0.0739	Shrubland	13.7	498	7.24	Spain	NA
	d1ba8f189fd7633e53a4d8cb109edcf1	ITS2	7940	ITS86F/ITS4	-0.5990°	39.8100°	Rhizosphere soil	4	180300	0.0221	Shrubland	13.7	498	7.24	Spain	NA
	ec0c41ce1a81fc8e77dd3837ab5b4c5e	ITS2	7940	ITS86F/ITS4	-0.5990°	39.8100°	Rhizosphere soil	4	180300	0.0221	Shrubland	13.7	498	7.24	Spain	NA
	f5b27a0dbb46b18d33735c0e873a9e26	ITS2	4008	ITS86F/ITS4	-0.5990°	39.8100°	Rhizosphere soil	13	175789	0.0739	Shrubland	13.7	498	7.24	Spain	NA
Malbranchea umbrina	48cfa647b43d6eaeb02300620628ca72	ITS1	3660	ITS1F/ITS4	143.9780°	-39.6652°	Soil	5223	168024	31.0848	Grassland	13.6	902	6	Australia	NA
	63c5681915266e6894bc6c8c67052a03	ITS1	3375	ITS1F/ITS4	147.2060°	-42.5591°	Soil	394	183462	2.1475	Grassland	11	621	6.3	Australia	NA
	268a87412a6732d93ec3895f89f13601	ITS1	3660	ITS1F/ITS4	143.9780°	-39.6652°	Soil	5223	168024	31.0848	Grassland	13.6	902	6	Australia	NA
	05e868cda13b07e391e67670a28c8154	ITS1	3660	ITS1F/ITS4	143.9780°	-39.6652°	Soil	5223	168024	31.0848	Grassland	13.6	902	6	Australia	NA
	aa0058509589bdec8803d7807f8b0694	ITS1	30507	ITS1ngs/ITS4ngs	23.0754°	58.5970°	Soil	1	3067	0.3260	Grassland	6.7	598	NA	Estonia	*Platanthera chlorantha*
	1bc6a3e669e2a15c2e04d0612ca1d668	ITS1	3375	ITS1F/ITS4	147.2060°	-42.5591°	Soil	394	183462	2.1475	Grassland	11	621	6.3	Australia	NA
	1c6e10293ec4f4db86091c8262460fa7	ITS1	88274	ITS9MUNngs/ITS4ngsUni	14.2031°	50.5590°	Soil	15	14241	1.0532	Forest	9	520	NA	Czech Republic	NA
	2c1121416373432b5dc8eb1c5ee60074	ITS1	3660	ITS1F/ITS4	143.9780°	-39.6652°	Soil	5223	168024	31.0848	Grassland	13.6	902	6	Australia	NA
	66258fde92ed296945e28d055369d979	ITS1	153	ITS1F/ITS4	143.9730°	-39.6509°	Soil	3962	229833	17.2386	Grassland	13.6	898	6.3	Australia	NA
	a5d82710dd8f13a6a93f301d13d9f33b	ITS1	6360	ITS1F/ITS4	143.9780°	-39.6652°	Soil	5223	168024	31.0848	Grassland	13.6	902	6	Australia	NA
	55a9b72ff76876945ceafbdbf4fcef81	ITS2	32534	fITS9/ITS4	23.2006°	41.6661°	Soil	3	5378	0.5578	Grassland	14	471	7.62	Grece	*Tragopogon dubius*
	0bbc76d8305028322152d0f89d594ea0	ITS2	2750	ITS3-Mix1 to 2/ITS4-cwmix1 + ITS4-cwmix2	11.9304°	57.6214°	Soil	1	210391	0.0047	Grassland	8	801	6.13	Sweden	*Anthriscus sylvestris*
	c681c34747874919975586c6e40c0843	ITS2	12821	fITS7/ITS4	143.9730°	-39.6509°	Soil	1	69976	0.0142	Forest	13.6	898	6.3	Australia	NA
	a65b4142d6e9b6c348f33ce73f7282f4	ITS2	8993	ITS9MUNngs/ITS4ngsUni	14.1742°	49.5002°	Soil	3	7710	0.3891	Forest	8.5	513	NA	Czech Republic	NA
	37cbcadce36c48343a7230eb7344f106	ITS2	13814	ITS9MUNngs/ITS4ngsUni	14.1742°	49.5002°	Soil	6	21584	0.2779	Forest	8.5	513	NA	Czech Republic	NA
	6f8eb23c8585c4777bb61a30a308dc0f	ITS2	13814	ITS9MUNngs/ITS4ngsUni	14.1742°	49.5002°	Soil	6	21584	0.2779	Forest	8.5	513	NA	Czech Republic	NA
Myriodontium keratinophilum	b632cc842e33fe1191ca5be218bf55cd	ITS1	6989	ITS3_KYO02/ITS4	-2.8696°	42.5867°	Root	61	109759	0.5557	Cropland	13	601	8.1	Spain	*Vitis vinifera*
	8112b4833379faaf92bfc523fd634d84	ITS1	9584	ITS3_KYO02/ITS4	-2.8528°	42.5928°	Rhizosphere soil	17	111334	0.1526	Cropland	13	598	8.2	Spain	*Vitis vinifera*
	013598c790b9b858a456176155b7ecd0	ITS1	6989	ITS3_KYO02/ITS4	-2.8695°	42.5867°	Root	61	109759	0.5557	Cropland	13	601	8.1	Spain	*Vitis vinifera*
	2a4b62c85b06647fefec737e2a80304a	ITS1	261	ITS1F/ITS4	138.1210°	-35.6364°	Soil	99	246488	0.4016	Woodland	15.5	632	8.5	Australia	NA

	5364e263a98c9b64def91949f6ef3549	ITS1	90126	ITS1F/ITS4	138.1210°	-35.6348°	Soil	28	151241	0.1851	Woodland	15.5	632	8.2	Australia	NA
	e69a59d6a36bc41d9ed65ab7d5b8851a	ITS1	3558	ITS1F/ITS4	138.6920°	-34.7735°	Soil	50	136733	0.3656	Woodland	15.4	614	7.83	Australia	NA
	6fdba33be67cf8cea1e5fd0aff792733	ITS1	996	ITS1F/ITS4	138.1260°	-35.6382°	Soil	3	237916	0.0126	Woodland	15.3	673	5.9	Australia	NA
	9c6cd8c46298f0af734c3efc617fb93a	ITS1	261	ITS1F/ITS4	138.1210°	-35.6364°	Soil	99	246488	0.4016	Woodland	15.5	632	8.5	Australia	NA
	842f6acf69311d0952307039e7633ec0	ITS1	261	ITS1F/ITS4	138.1210°	-35.6364°	Soil	99	246488	0.4016	Woodland	15.5	632	8.5	Australia	NA
	fc13b6d20bbe7a8352a5363b2b963364	ITS1	12850	ITS5/ITS2	116.4800°	39.8800°	Air	1	52993	0.0188	Urban	12.6	609	NA	China	NA
	ac48708b22c62cea2d0b84f6c16f3cfd	ITS2	383	ITS1F/ITS4	147.3880°	-33.8449°	Soil	284	36251	7.8342	Urban	NA	NA	NA	Australia	NA
	b6b27131119016b221d45fd91a985313	ITS2	14295	ITS86F/ITS4	147.3880°	-33.8449°	Soil	78	142819	0.5461	Woodland	NA	NA	NA	Australia	NA
	8334fb694d4569002e570ce461d8fdb1	ITS2	7529	ITS1F/ITS4	147.3880°	-33.8449°	Soil	4	161891	0.0247	Woodland	NA	NA	NA	Australia	NA
	75d4348ddf11e3426da76513adf65655	ITS2	383	ITS1F/ITS4	147.3880°	-33.8449°	Soil	284	36251	7.8342	Urban	NA	NA	NA	Australia	NA
	6eea9c9203e740c9a55fe52890a04d6c	ITS2	20796	ITS1F/ITS4	147.3880°	-33.8449°	Soil	5	52598	0.0950	Urban	NA	NA	NA	Australia	NA
	246be542e5056e2ee90da0cca2c2811b	ITS2	7529	ITS1F/ITS4	147.3880°	-33.8449°	Soil	4	161891	0.0247	Woodland	NA	NA	NA	Australia	NA
	1693fa65e00d3561d6c5c74ba2eff2b5	ITS2	383	ITS1F/ITS4	147.3880°	-33.8449°	Soil	284	36251	7.8342	Urban	NA	NA	NA	Australia	NA
	7bc0310bc55364cb5e40774a87265b3f	ITS2	3486	ITS86F/ITS4	-2.85282°	42.5928°	Rhizosphere soil	78	142819	0.5461	Shrubland	NA	NA	NA	Spain	NA
	7bc0310bc55364cb5e40774a87265b3f|	ITS2	383	ITS1F/ITS4	147.3880°	-33.8449°	Soil	284	36251	7.8342	Urban	NA	NA	NA	Australia	NA
	ce0448ca2d562aee1cec131f0f2e7708	ITS2	3486	ITS86F/ITS4	-2.8528°	42.5928°	Rhizosphere soil	78	142819	0.5461	Shrubland	NA	NA	NA	Spain	NA
Neoarthropsis hispanica	4cc4104c05180c30c5d8002ad2cd05d5	ITS1	3066	ITS1F/ITS2	143.1440°	-37.3155°	Soil	350	67725	5.1679	Cropland	12.9	547	6.7	Australia	*Vitis vinifera*
	65bf880175c024cf31d6dcd69e66e8e5	ITS1	35783	ITS5/ITS2	106.5700°	26.6600°	Soil	3	78503	0.0382	Cropland	15.1	9.21	7.32	China	*Zea mays*
	40cf2e717653ab7099923e7250254e1c	ITS1	35783	ITS5/ITS2	106.5700°	26.6600°	Soil	3	78503	0.0382	Cropland	15.1	9.21	7.32	China	*Zea mays*
	edd1864aeecfeec794949907032a4054	ITS1	19084	ITS5/ITS2	106.5700°	26.6600°	Soil	325	57018	5.6999	Forest	NA	NA	NA	China	NA
	1fec869b64ba2f1a2c553af845dc382a	ITS1	6176	ITS1F/ITS4	105.5570°	-10.4788°	Soil	16	185285	0.0863	Forest	26.4	1772	7	Australia	NA
	01aea6da4950e4538494b1d7888f8baf	ITS1	6176	ITS1F/ITS4	105.5570°	-10.4788°	Soil	16	185285	0.0863	Forest	26.4	1772	7	Australia	NA
	942c0d9132e722d33218633189c7510f	ITS1	31064	ITS5/ITS2	116.4800°	39.8800°	Air	1	53138	0.0188	Urban	12.6	609	NA	China	NA
	69c8d0fbaadb1261a386813f9acd0da7	ITS1	3101	ITS1F/ITS4	126.9550°	37.4653°	Air	4	71226	0.0561	Urban	12	1383	NA	South Korea	NA
	be5b8960de60a541376e2fdd2eb4c371	ITS1	6176	ITS1F/ITS4	105.5570°	-10.4788°	Soil	16	185285	0.0863	Forest	26.4	1772	7	Australia	NA
	a33435a4c1e6f39dfa7f405b75632e08	ITS1	34826	ITS9MUNngs/ITS4ngsUni	26.6224°	59.4217°	Soil	2	8525	0.2346	Urban	6	599	NA	Estonia	NA
	aad89f84073c6ebcd8bd13891b38fe0d	ITS2	5691	fITS7/ITS4	114.6500°	22.5700°	Root	212	42341	5.0069	Shrubland	23.2	1929	NA	China	*Mussaenda kwangtungensis*
	c1b37851a02fddf789a908294e4eb5cd	ITS2	19349	fITS7/ITS4	114.6500°	22.5700°	Rhizosphere soil	133	62195	2.1384	Shrubland	23.2	1929	NA	China	*Mussaenda kwangtungensis*
	a69ab1f930a9fa38690a88a6640e687a	ITS2	17538	gITS7/ITS4	108.7830°	18.7000°	Soil	85	37901	2.2426	Forest	24.7	1196	6.7	China	*Castanopsis carlesii*
	9a96ecd907c063cb6be4b0f22dc996d3	ITS2	1401	ITS3_KYO2/ITS4	113.7760°	35.1278°	Rhizosphere soil	17	3516	4.8350	Cropland	15	586	7.15	China	*Malus robusta Rehd*
	5de791713be1cefce6da52897bba5f53	ITS2	19349	fITS7/ITS4	114.6500°	22.5700°	Rhizosphere soil	133	62195	2.1384	Shrubland	23.2	1929	NA	China	*Mussaenda kwangtungensis*
	43781e469b92027c20542855f1d5da91	ITS2	24367	ITS3_KYO2/ITS4	113.7660°	35.1278°	Rhizosphere soil	61	37248	1.6376	Cropland	15	586	7	China	*Punica granatum*
	3ef9835296ea904553cffed4f958643c	ITS2	3313	ITS1F/ITS4	138.1160°	-35.6327°	Soil	52	157047	0.3311	Woodland	15.5	639	6	Australia	NA
	e907ac06a7292f99155d2442c8ffb927	ITS2	22622	ITS3_KYO2/ITS4	113.7660°	35.1278°	Rhizosphere soil	101	47605	2.1216	Cropland	15	586	7	China	NA
	cecc282b6306dd698c13cb02042a8fe2	ITS2	26147	fITS7/ITS4	112.3330°	16.8333°	Soil	23	28782	0.7991	Woodland	26.8	1504	8.71	China	NA
	fd477cc21b5237f552a04492512cb152	ITS2	16692	ITS9/ITS4	132.9160°	34.2978°	Sediment	276	51771	5.3311	Aquatic	16.9	1288	NA	Japan	NA

Neoarthropsis sexualis	8185dcee25c105bfb0b2f0e3662264a4	ITS1	34428	ITS5/ITS2	26.6224°	59.4217°	Soil	9	34480	0.2610	Grassland	NA	NA	NA	Estonia	NA
	7255587fd47511e70b2d96f92082a771	ITS1	2055	ITS1F/ITS4	138.1210°	-35.6364°	Soil	1	24648	0.0405	Grassland	15.5	632	8.5	Australia	NA
	68b299cb1d77766e47660959035767db	ITS1	40897	ITS5/ITS2	26.6224°	59.4217°	Soil	9	34480	0.2610	Grassland	NA	NA	NA	Estonia	NA
	7fb168f5a0f149dae418bdcaf2d543fa	ITS2	8283	gITS7ngs/ITS4ngsUni	-111.5680°	35.5722°	Soil	1	282837	0.0035	Forest	NA	NA	NA	USA	NA
	ac3b461583e8ee39d91cdf9ca19d9797	ITS2	1430	gITS7ngs/ITS4ngsUni	-111.5680°	35.5722°	Soil	1	314307	0.0031	Forest	NA	NA	NA	USA	NA
	4b8caf103c5d55836a7944a55fa398b7	ITS2	5388	gITS7ngs/ITS4ngsUni	-111.5680°	35.5722°	Soil	5	153897	0.0324	Forest	NA	NA	NA	USA	NA
	7b0dad2499764e751c26de620e73a14f	ITS2	16552	gITS7ngs/ITS4ngsUni	-111.5680°	35.5722°	Soil	3	234630	0.0127	Forest	NA	NA	NA	USA	NA
	2589891256c1a648275483f8a49d5321	ITS2	24795	gITS7ngs/ITS4ngsUni	-111.5680°	35.5722°	Soil	1	391156	0.0025	Forest	NA	NA	NA	USA	NA
	2060fd663829fddbaaca1f510491775e	ITS2	16552	gITS7ngs/ITS4ngsUni	-111.5680°	35.5722°	Soil	3	234630	0.0127	Forest	NA	NA	NA	USA	NA
	9bfe26c65f8ca1bdb11becfa49157398	ITS2	16552	gITS7ngs/ITS4ngsUni	-111.5680°	35.5722°	Soil	3	234630	0.0127	Forest	NA	NA	NA	USA	NA
Pseudoamaurascopsis spiralis	b00bda826c9f9b2dcb557f576caf2f3b	ITS1	26312	ITS1F/58A2R	9.81922°	56.5354°	Soil	4	5596	0.7147	Cropland	8.3	618	5.9	Denmark	*Secale* sp.
	ba41b4f3b3a33c3daf3c52fc0023b25e	ITS1	18948	ITS1F/58A2R	9.81922°	56.5354°	Soil	11	1899	5.7925	Cropland	8.3	618	5.9	Denmark	*Secale* sp.
	7857d46e6e384677180caf3073f29816	ITS1	33069	ITS1/ITS4	125.2170°	49.1167°	Soil	52	18796	2.7665	Cropland	1.7	493	5.32	China	*Glycine* sp.
	4f32c6dffc49fb20737038fbf484752e	ITS1	6783	ITS5/5.8S_fungi	-5.27019°	51.7000°	Soil	37	524903	0.0704	Grassland	11	997	5.2	UK	NA
	3ec399c5f82f86e41d7e1da82503ed57	ITS1	31840	ITS1/ITS4	126.1330°	48.8667°	Soil	11	14022	0.7844	Cropland	1.2	523	5.39	China	*Glycine* sp.
	884d3adc4ec14e1018e7b65eb6dbcd4d	ITS1	11441	ITS5/5.8S_fungi	-3.1097°	52.6375°	Soil	5722	227200	25.1848	Grassland	9.1	1218	5.2	UK	NA
	4be2c89cb967bb9cdfd6bcfde9a19548	ITS1	14940	ITS3_KYO2/ITS4	-2.7738°	42.5406°	Root	857	153279	5.5911	Cropland	13	626	8.2	Spain	*Vitis vinifera*
	5f0a38522aef314efb5cd66ee746b313	ITS1	11441	ITS5/5.8S_fungi	-3.1097°	52.6375°	Root	5722	227200	25.1848	Grassland	9.1	1218	5.2	UK	NA
	80c894e5fddd940df70aea933a4098bc	ITS1	9584	ITS3_KYO2/ITS4	-2.8528°	42.5928°	Rhizosphere soil	151	111334	1.3562	Cropland	13	598	8.2	Spain	*Vitis vinifera*
	c44d6cbd333d56b5dffbb010ede59f87	ITS1	68233	ITS9MUNngs/ITS4ngsUni	25.6823°	58.1761°	Soil	1	5129	0.1949	Urban	5.4	738	NA	Estonia	NA
	f7363f680630637ff0abdcc37e61da28	ITS1	699	ITS1F/ITS4	149.5980°	-30.2007°	Soil	22	172977	0.1271	Cropland	19.3	600	7.3	Australia	NA
	0ae8874645603a0a1b3449fd635c6e20	ITS1	14940	ITS3_KYO2/ITS4	-2.7738°	42.5406°	Root	857	153279	5.5911	Cropland	13	626	8.2	Spain	*Vitis vinifera*
	0c3c6f8057a971cd017f76afff0b0149	ITS1	11441	ITS5/5.8S_fungi	-3.1097°	52.6375°	Soil	5722	227200	25.1848	Grassland	9.1	1218	5.2	UK	NA
	3ff68c27a9f6b92c0ece003bef060a34	ITS1	11441	ITS5/5.8S_fungi	-3.1097°	52.6375°	Soil	5722	227200	25.1848	Grassland	9.1	1218	5.2	UK	NA
	98862b05554d102f490d20ac87d036df	ITS1	11441	ITS5/5.8S_fungi	-3.1097°	52.6375°	Soil	5722	227200	25.1848	Grassland	9.1	1218	5.2	UK	NA
	55c448788fe0a0fa89072522a5b6e997	ITS1	29417	ITS3_KYO2/ITS4	-2.8528°	42.5803°	Rhizosphere soil	1375	145254	9.4661	Cropland	13.1	602	8.1	Spain	*Vitis vinifera*
	ce07c48dee18aa33bddc8ea83536bfb5	ITS1	11441	ITS5/5.8S_fungi	-3.1097°	52.6375°	Soil	5722	227200	25.1848	Grassland	9.1	1218	5.2	UK	NA
	d79d1f153361d021bd226531ec9d8815	ITS1	14940	ITS3_KYO2/ITS4	-2.7738°	42.5406°	Root	857	153279	5.5911	Cropland	13	626	8.2	Spain	*Vitis vinifera*
	212d253464c4575145ad1a7d2e7c70b2	ITS1	11441	ITS5/5.8S_fungi	-3.1097°	52.6375°	Root	5722	227200	25.1848	Grassland	9.1	1218	5.2	UK	NA
	80601a9a574b822537bef70ad03d1b02	ITS1	14940	ITS3_KYO2/ITS4	-2.7738°	42.5406°	Root	857	153279	5.5911	Cropland	13	626	8.2	Spain	*Vitis vinifera*
	f226b5efb0204f485a373dfa46486514	ITS2	4832	fITS9/ITS4	-89.3500°	43.1333°	Soil	68	137865	0.4932	Grassland	8.7	775	6.35	USA	*Panicum virgatum*
	b15daf29f8118851bd841fa664a68049	ITS2	18872	ITS86F/ITS4	124.2330°	43.3167°	Soil	228	60579	3.7636	Cropland	7	569	6.95	China	*Zea mays*
	37fa421de98fa0e98a0d844ac8074ae9	ITS2	23749	ITS86F/ITS4	124.2330°	43.3167°	Soil	268	46501	5.7633	Cropland	7	569	6.95	China	*Zea mays*
	438e8a013caac8741dafba7405014dfa	ITS2	16460	ITS3ngs mix/ITS4ngs	3.5325°	50.5083°	Soil	34	142203	0.2390	Cropland	10.7	758	5.3	Belgium	*Triticum* sp.
	4da2830e5e269b41a50c1d0a57f20435	ITS2	16326	ITS1F/ITS4	149.5970°	-30.2012°	Soil	69	171162	0.4031	Cropland	19.3	600	7.2	Australia	NA
	7b6c0de1b81efc5b48faedb8ea67b5f0	ITS2	33968	ITS86F/ITS4	124.2330°	43.3167°	Soil	325	54260	5.9896	Cropland	7	569	6.95	China	*Zea mays*

93cf081c95521d6d7f9248e32a55ad33	ITS2	9954	fITS9/ITS4	8.7719°	50.2717°	Soil	1	5166	0.1935	Grassland	10.3	588	6.5	Germany	*Geranium molle*
a79a2b87879ab5a20b789ec70ebdb1e9	ITS2	5905	fITS9/ITS4	-85.3700°	42.3800°	Soil	598	183795	3.2536	Grassland	9.2	881	6.35	USA	*Panicum virgatum*
bfd8a04230953813b7d6605244f58716	ITS2	3293	ITS86F/ITS4	124.2330°	43.3167°	Soil	201	59989	3.3506	Cropland	7	569	6.9	China	*Zea mays*
b7934349623461d77b89c638a0b0f56f	ITS2	11717	ITS86F/ITS4	124.2330°	43.3167°	Soil	161	56825	2.8332	Cropland	7	569	6.9	China	*Zea mays*

[1]Internal Transcribed Spacer region; [2] Number assigned to a particular environmental sample; [3]Abundance of a singular sequence in a particular environmental sample; [4]Total abundance of ITS sequences found in a particular environmental sample; [5]Relative abundance of a singular ITS sequence in a particular environmental sample; [6]Mean Annual Temperature (ºC); [7]Mean Annual Precipitation (mm).

Figure S1.

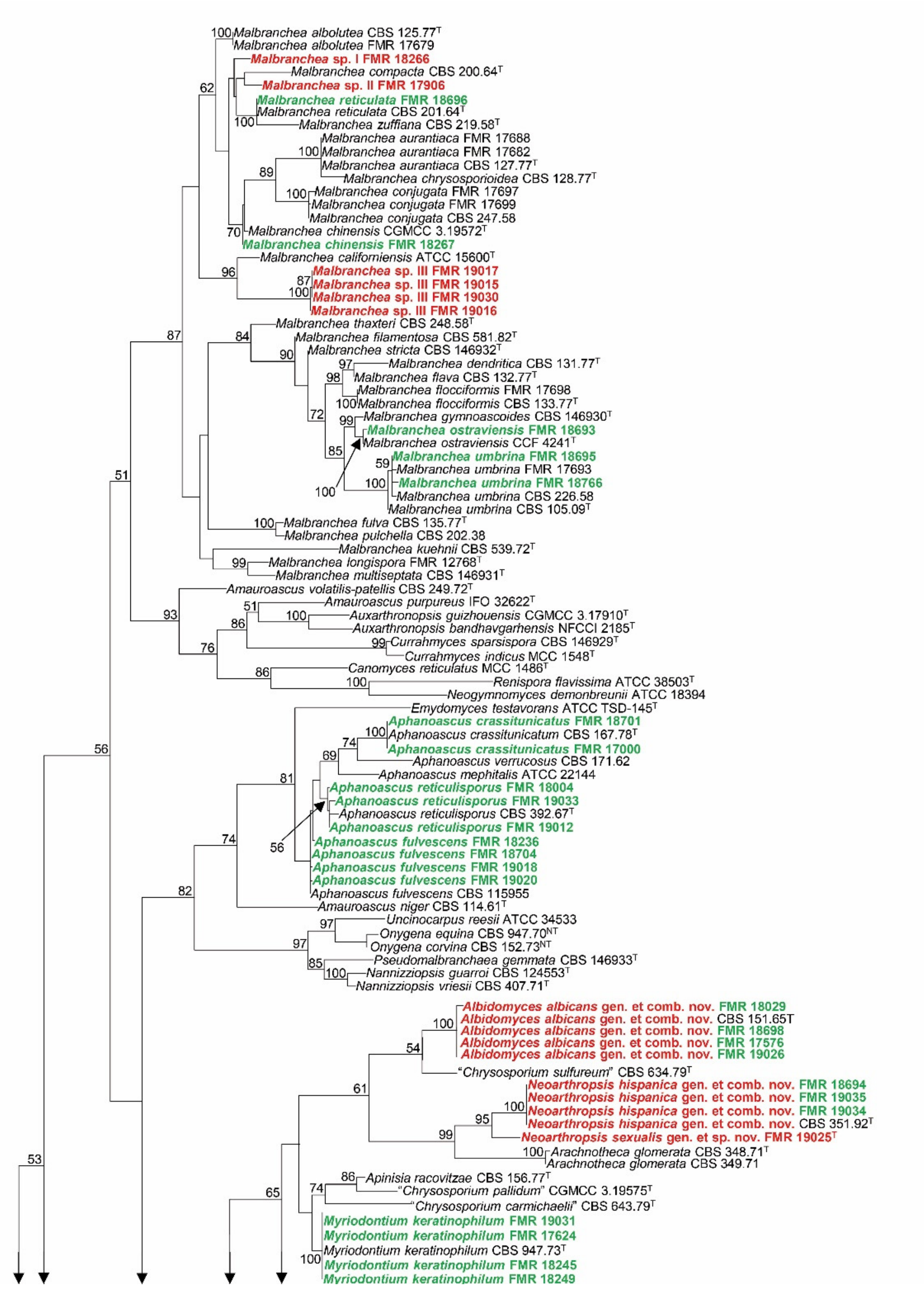

Malbranchea albolutea CBS 125.77T
Malbranchea albolutea FMR 17679
Malbranchea sp. I FMR 18266
Malbranchea compacta CBS 200.64T
Malbranchea sp. II FMR 17906
Malbranchea reticulata FMR 18696
Malbranchea reticulata CBS 201.64T
Malbranchea zuffiana CBS 219.58T
Malbranchea aurantiaca FMR 17688
Malbranchea aurantiaca FMR 17682
Malbranchea aurantiaca CBS 127.77T
Malbranchea chrysosporioidea CBS 128.77T
Malbranchea conjugata FMR 17697
Malbranchea conjugata FMR 17699
Malbranchea conjugata CBS 247.58
Malbranchea chinensis CGMCC 3.19572T
Malbranchea chinensis FMR 18267
Malbranchea californiensis ATCC 15600T
Malbranchea sp. III FMR 19017
Malbranchea sp. III FMR 19015
Malbranchea sp. III FMR 19030
Malbranchea sp. III FMR 19016
Malbranchea thaxteri CBS 248.58T
Malbranchea filamentosa CBS 581.82T
Malbranchea stricta CBS 146932T
Malbranchea dendritica CBS 131.77T
Malbranchea flava CBS 132.77T
Malbranchea flocciformis FMR 17698
Malbranchea flocciformis CBS 133.77T
Malbranchea gymnoascoides CBS 146930T
Malbranchea ostraviensis FMR 18693
Malbranchea ostraviensis CCF 4241T
Malbranchea umbrina FMR 18695
Malbranchea umbrina FMR 17693
Malbranchea umbrina FMR 18766
Malbranchea umbrina CBS 226.58
Malbranchea umbrina CBS 105.09T
Malbranchea fulva CBS 135.77T
Malbranchea pulchella CBS 202.38
Malbranchea kuehnii CBS 539.72T
Malbranchea longispora FMR 12768T
Malbranchea multiseptata CBS 146931T
Amauroascus volatilis-patellis CBS 249.72T
Amauroascus purpureus IFO 32622T
Auxarthronopsis guizhouensis CGMCC 3.17910T
Auxarthronopsis bandhavgarhensis NFCCI 2185T
Currahmyces sparsispora CBS 146929T
Currahmyces indicus MCC 1548T
Canomyces reticulatus MCC 1486T
Renispora flavissima ATCC 38503T
Neogymnomyces demonbreunii ATCC 18394
Emydomyces testavorans ATCC TSD-145T
Aphanoascus crassitunicatus FMR 18701
Aphanoascus crassitunicatum CBS 167.78T
Aphanoascus crassitunicatus FMR 17000
Aphanoascus verrucosus CBS 171.62
Aphanoascus mephitalis ATCC 22144
Aphanoascus reticulisporus FMR 18004
Aphanoascus reticulisporus FMR 19033
Aphanoascus reticulisporus CBS 392.67T
Aphanoascus reticulisporus FMR 19012
Aphanoascus fulvescens FMR 18236
Aphanoascus fulvescens FMR 18704
Aphanoascus fulvescens FMR 19018
Aphanoascus fulvescens FMR 19020
Aphanoascus fulvescens CBS 115955
Amauroascus niger CBS 114.61T
Uncinocarpus reesii ATCC 34533
Onygena equina CBS 947.70NT
Onygena corvina CBS 152.73NT
Pseudomalbranchaea gemmata CBS 146933T
Nannizziopsis guarroi CBS 124553T
Nannizziopsis vriesii CBS 407.71T
Albidomyces albicans gen. et comb. nov. FMR 18029
Albidomyces albicans gen. et comb. nov. CBS 151.65T
Albidomyces albicans gen. et comb. nov. FMR 18698
Albidomyces albicans gen. et comb. nov. FMR 17576
Albidomyces albicans gen. et comb. nov. FMR 19026
"Chrysosporium sulfureum" CBS 634.79T
Neoarthropsis hispanica gen. et comb. nov. FMR 18694
Neoarthropsis hispanica gen. et comb. nov. FMR 19035
Neoarthropsis hispanica gen. et comb. nov. FMR 19034
Neoarthropsis hispanica gen. et comb. nov. CBS 351.92T
Neoarthropsis sexualis gen. et sp. nov. FMR 19025T
Arachnotheca glomerata CBS 348.71T
Arachnotheca glomerata CBS 349.71
Apinisia racovitzae CBS 156.77T
"Chrysosporium pallidum" CGMCC 3.19575T
"Chrysosporium carmichaelii" CBS 643.79T
Myriodontium keratinophilum FMR 19031
Myriodontium keratinophilum FMR 17624
Myriodontium keratinophilum CBS 947.73T
Myriodontium keratinophilum FMR 18245
Myriodontium keratinophilum FMR 18249

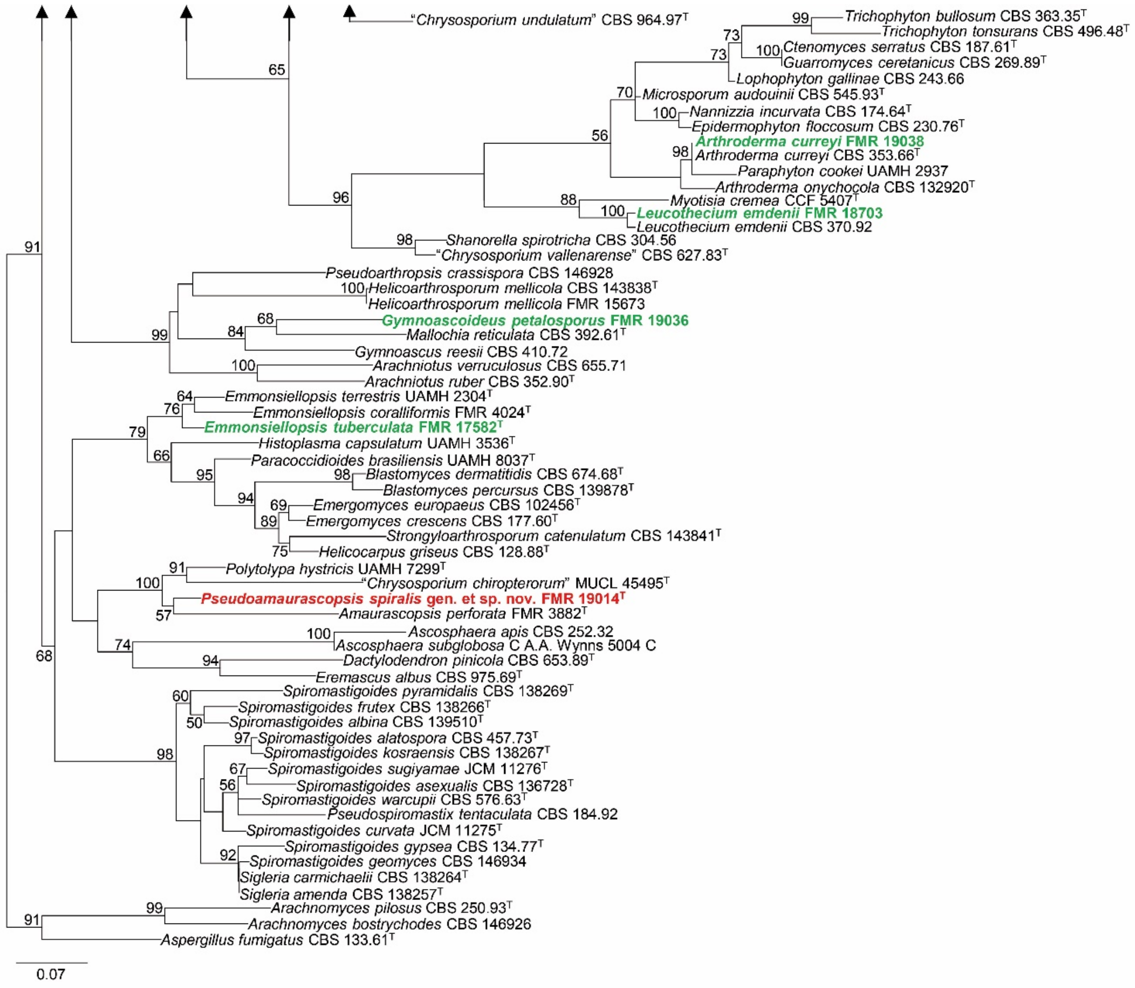

Figure S1. RAxML ITS tree of the order *Onygenales*, including the strains recovered from freshwater sediments studied in this work. The strains belonging to undescribed species or genera are in red and in bold. The strains belonging to knonw species are in green and in bold. Determined by MEGA software v.6, the best nucleotide substitution model for ML analysis was K2+G+I. The aligned data set was 598 bp long, with 440 variable sites and 373 phylogenetically informative. Branch lengths are proportional to phylogenetic distance. Bootstrap suport values above 50% are indicated on the nodes. The tree was rooted to *Arachnomyces pilosus* CBS 250.93, *Arachnomyces bostrychodes* CBS 146926, and *Aspergillus fumigatus* CBS 133.61. Quote marks indicate species with unresolved taxonomy. ^{T}= Ex-type strain.

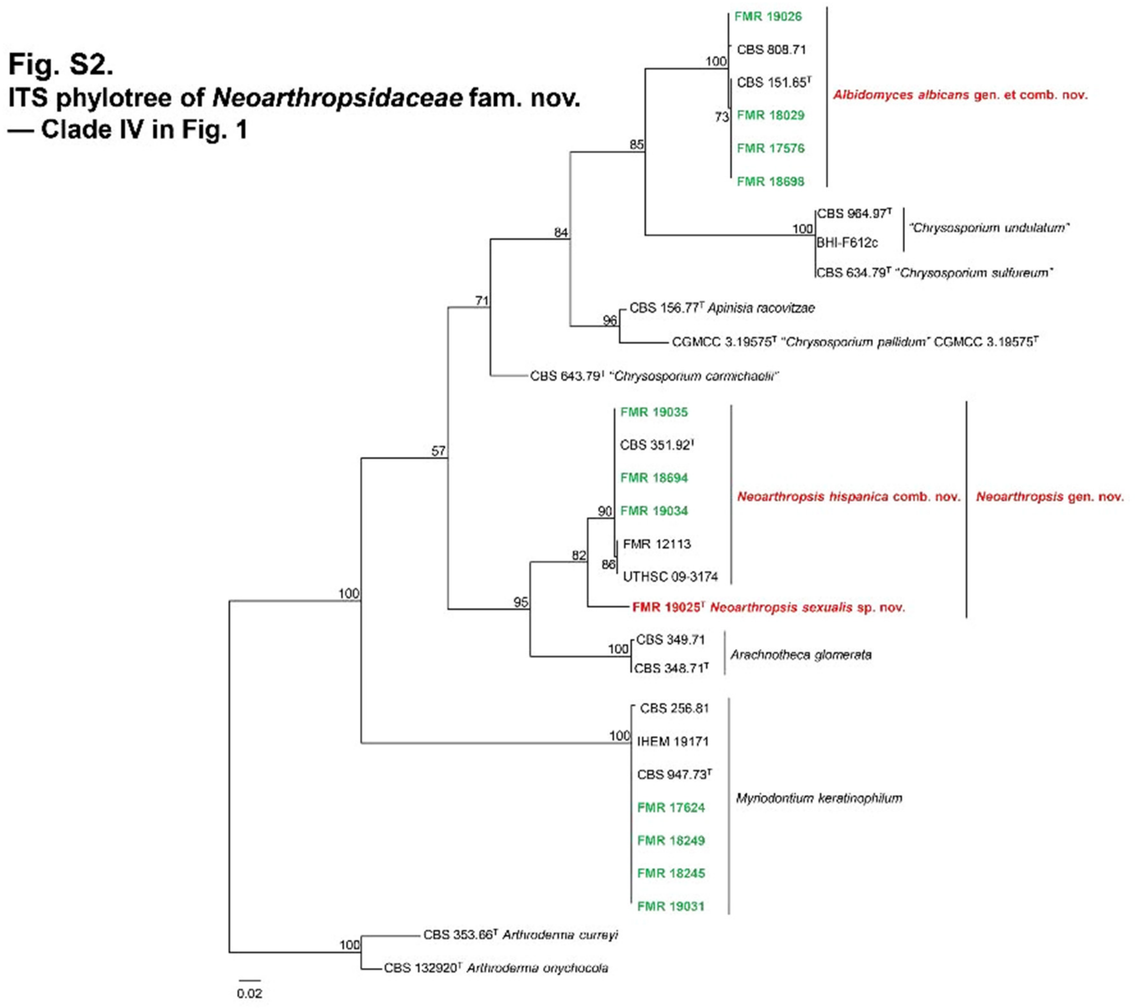

Figure S2. RAxML ITS tree of the family *Neoarthropsidaceae*, including the strains recovered from freshwater sediments studied in this work. New taxa proposed are in red and in bold; strains of known species identified in our study are in green. Determied by MEGA software v.6., the best nucleotide substitution model for ML analysis was K2+G. The aligned data set was 408 bp long, with 161 variable sites and 149 phylogenetically informative. Branch lengths are proportional to phylogenetic distances. Bootstrap suport values above 70% are indicated on the nodes. The tree was rooted to *Arthroderma curreyi* CBS 335.66 and *Arthroderma onychocola* CBS 132920. ^{T}= Ex-type strain.

Figure S3.

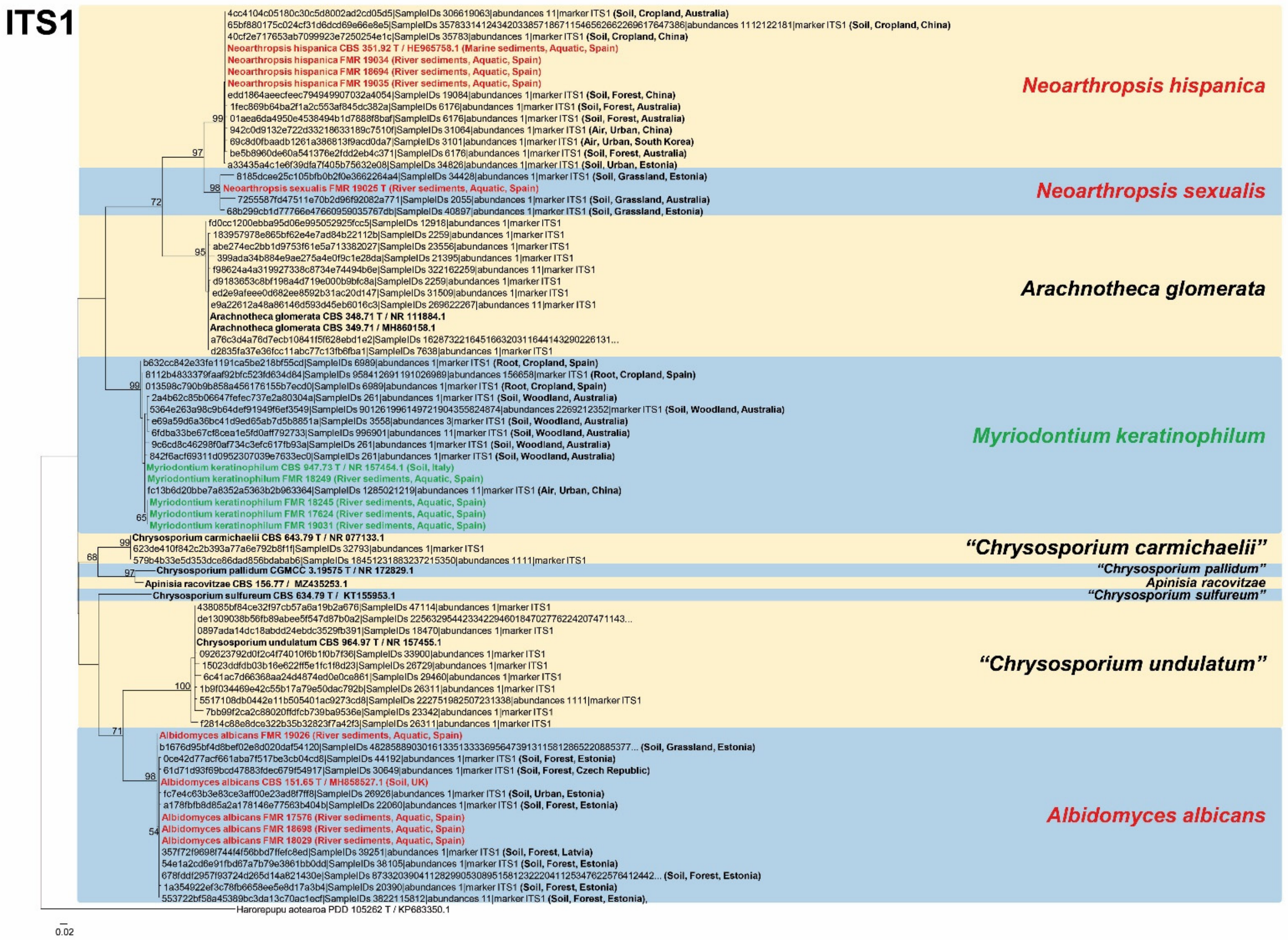

ITS1
Neoarthropsis hispanica
Neoarthropsis sexualis
Arachnotheca glomerata
Myriodontium keratinophilum
"Chrysosporium carmichaelii"
"Chrysosporium pallidum"
Apinisia racovitzae
"Chrysosporium sulfureum"
"Chrysosporium undulatum"
Albidomyces albicans
Harorepupu aotearoa PDD 105262 T / KP683350.1
0.02

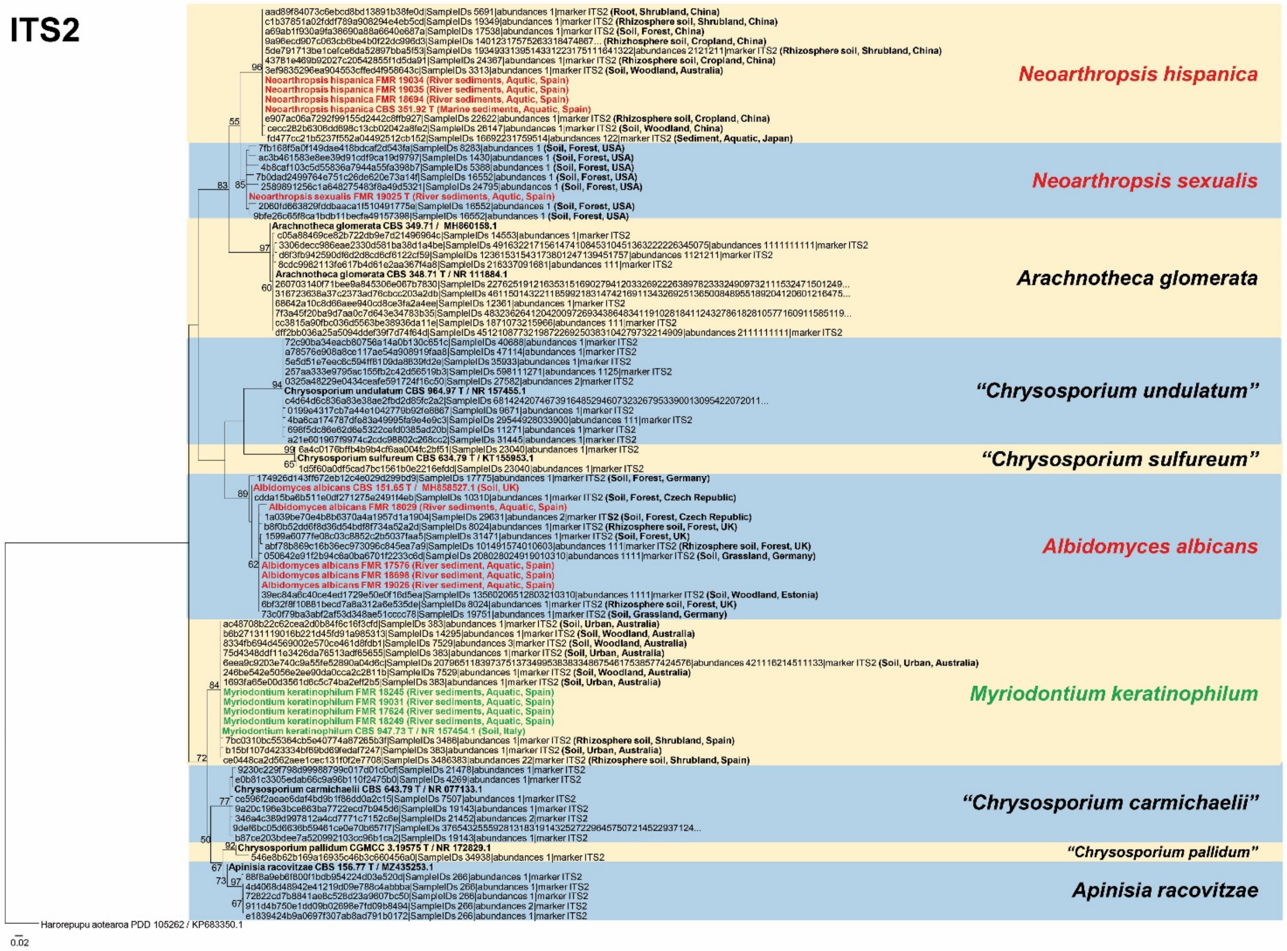

Figure S3. RAxML phylogenetic tree showing the relationships among species investigated in the new family *Neoarthropsidaceae,* and related ITS1/ITS2 environmental sequences deposited in the GlobalFungi database. Titles of sequences contain sequence and sample codes taken from GlobalFungi. ITS1/ITS2 sequences of novel taxa are in red. ITS1/ITS2 sequences of sediment isolates and ex-type strains of known species identified in this study are in green. ITS1/ITS2 sequences of known species retreived from GenBank are in bold. Geographical and ecological information contained in environmental sequences assocítated to the novel taxa are written in bold. Determined by MEGA software v.6., the best nucleotide substitution model for ML analysis was K2+G for ITS1 and JC+G for ITS2. The alignments data sets were 381 bp long for ITS1 and 181 bp for ITS2; with 228 variable sites for ITS1 and 127 for ITS2; and 167 phylogenetically informative for ITS1 and 97 for ITS2. Branch lengths are proportional to phylogenetic distances. Bootstrap support values above 50% are indicated on the nodes. The trees were rooted to *Harorepupu aotearoa* PDD 105262. Quote marks indicate species with unresolved taxonomy. ^{T}= Ex-type strain.

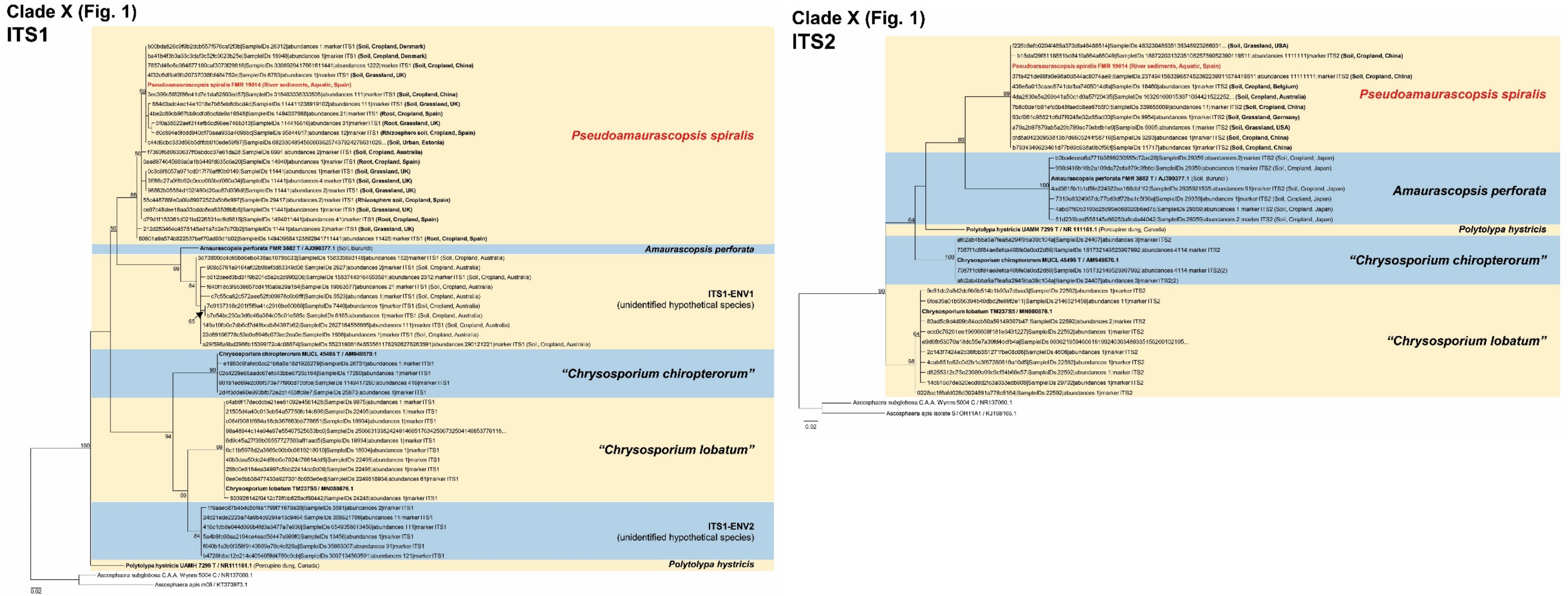

Figure S4. RAxML phylogenetic tree showing the relationships among species investigated in the Clade X (*incertae sedis* at the family level), and related ITS1/ITS2 environmental sequences deposited in the GlobalFungi database. Titles of sequences contain sequence and sample codes taken from GlobalFungi. ITS1/ITS2 sequence of the novel genus *Pseudoamaurascopsis* is in red. ITS1/ITS2 sequences of known species retreived from GenBank are in bold. Geographical and ecological information contained in environmental sequences assocytated to the novel genus are written in bold. Determined by MEGA software v.6., the best nucleotide substitution model for ML analysis was K2+G for ITS1 and T92 for ITS2. The alignments data sets were 208 bp long for ITS1 and 171 bp for ITS2; with 125 variable sites for ITS1 and 80 for ITS2; and 97 phylogenetically informative for ITS1 and 50 for ITS2. Branch lengths are proportional to phylogenetic distances. Bootstrap support values above 50% are indicated on the nodes. The trees were rooted to *Ascosphaera subglobosa* C.A.A. Wynns 5004 (C) and *Ascosphaera apis* m05. Quote marks indicate species with unresolved taxonomy. ^{T}= Ex-type strain.

Figure S5.

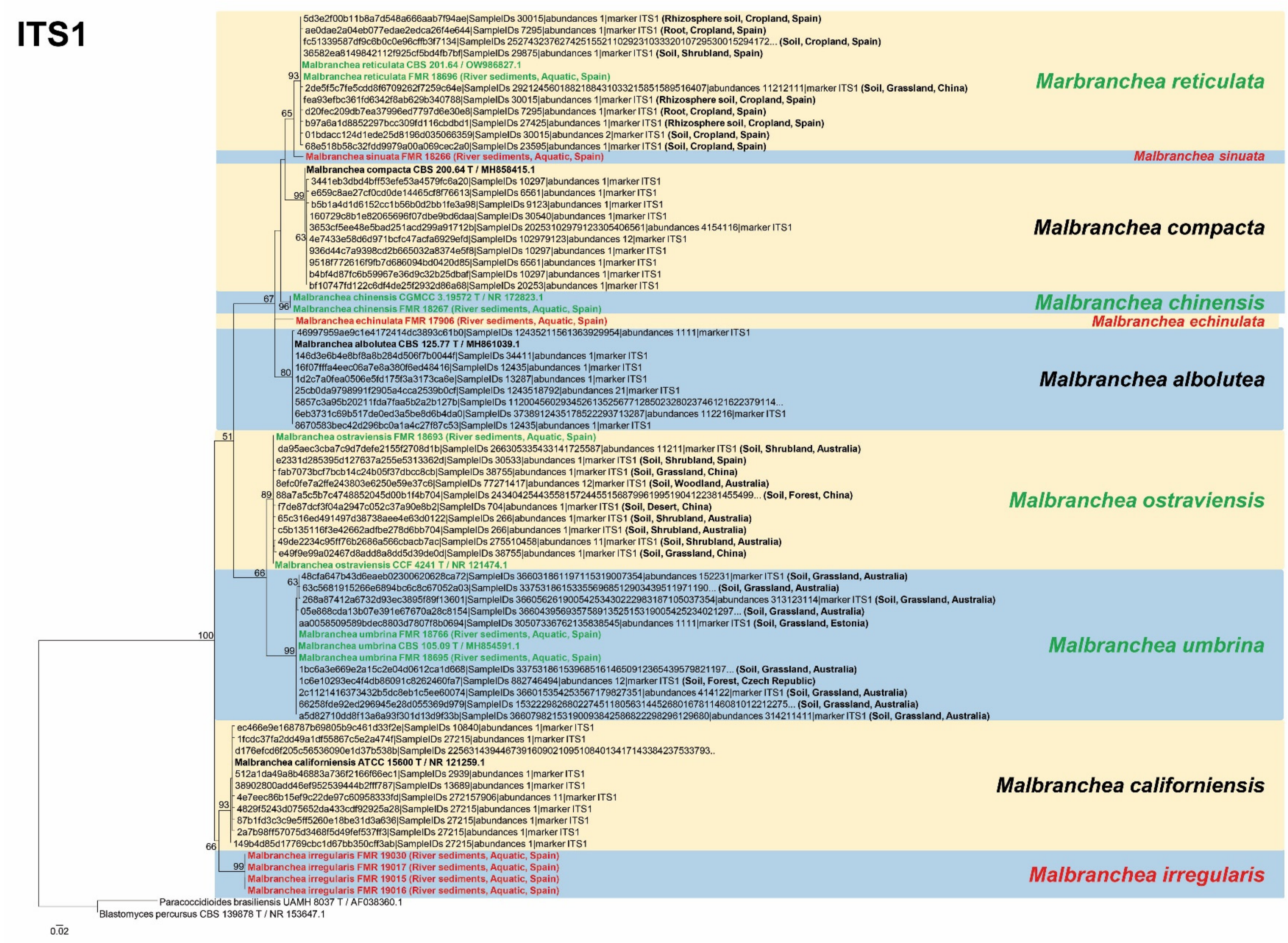
ITS1
Marbranchea reticulata
Malbranchea reticulata CBS 201.64 / OW986827.1
Malbranchea reticulata FMR 18696 (River sediments, Aquatic, Spain)
Malbranchea sinuata
Malbranchea sinuata FMR 18266 (River sediments, Aquatic, Spain)
Malbranchea compacta
Malbranchea compacta CBS 200.64 T / MH858415.1
Malbranchea chinensis
Malbranchea chinensis CGMCC 3.19572 T / NR 172823.1
Malbranchea chinensis FMR 18267 (River sediments, Aquatic, Spain)
Malbranchea echinulata
Malbranchea echinulata FMR 17906 (River sediments, Aquatic, Spain)
Malbranchea albolutea
Malbranchea albolutea CBS 125.77 T / MH861039.1
Malbranchea ostraviensis
Malbranchea ostraviensis FMR 18693 (River sediments, Aquatic, Spain)
Malbranchea ostraviensis CCF 4241 T / NR 121474.1
Malbranchea umbrina
Malbranchea umbrina FMR 18766 (River sediments, Aquatic, Spain)
Malbranchea umbrina CBS 105.09 T / MH854591.1
Malbranchea umbrina FMR 18695 (River sediments, Aquatic, Spain)
Malbranchea californiensis
Malbranchea californiensis ATCC 15600 T / NR 121259.1
Malbranchea irregularis
Malbranchea irregularis FMR 19030 (River sediments, Aquatic, Spain)
Malbranchea irregularis FMR 19017 (River sediments, Aquatic, Spain)
Malbranchea irregularis FMR 19015 (River sediments, Aquatic, Spain)
Malbranchea irregularis FMR 19016 (River sediments, Aquatic, Spain)
Paracoccidioides brasiliensis UAMH 8037 T / AF038360.1
Blastomyces percursus CBS 139878 T / NR 153647.1
0.02

Figure S5.

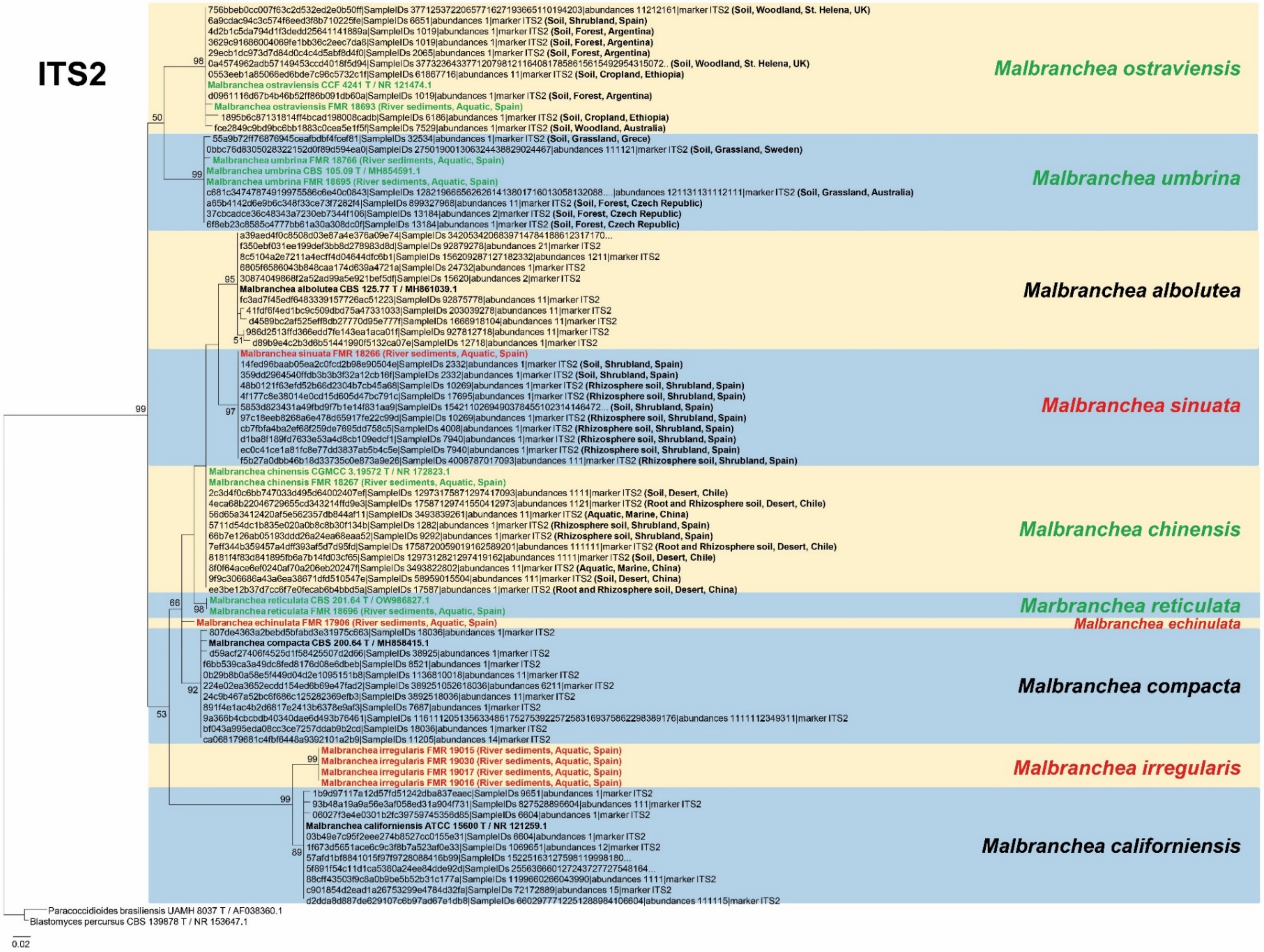

ITS2
Malbranchea ostraviensis
Malbranchea umbrina
Malbranchea albolutea
Malbranchea sinuata
Malbranchea chinensis
Marbranchea reticulata
Malbranchea echinulata
Malbranchea compacta
Malbranchea irregularis
Malbranchea californiensis
Malbranchea ostraviensis CCF 4241 T / NR 121474.1
Malbranchea ostraviensis FMR 18693 (River sediments, Aquatic, Spain)
Malbranchea umbrina FMR 18766 (River sediments, Aquatic, Spain)
Malbranchea umbrina CBS 105.09 T / MH854591.1
Malbranchea umbrina FMR 18695 (River sediments, Aquatic, Spain)
Malbranchea albolutea CBS 125.77 T / MH861039.1
Malbranchea sinuata FMR 18266 (River sediments, Aquatic, Spain)
Malbranchea chinensis CGMCC 3.19572 T / NR 172823.1
Malbranchea chinensis FMR 18267 (River sediments, Aquatic, Spain)
Malbranchea reticulata CBS 201.64 T / OW986827.1
Malbranchea reticulata FMR 18696 (River sediments, Aquatic, Spain)
Malbranchea echinulata FMR 17906 (River sediments, Aquatic, Spain)
Malbranchea compacta CBS 200.64 T / MH858415.1
Malbranchea irregularis FMR 19015 (River sediments, Aquatic, Spain)
Malbranchea irregularis FMR 19030 (River sediments, Aquatic, Spain)
Malbranchea irregularis FMR 19017 (River sediments, Aquatic, Spain)
Malbranchea irregularis FMR 19016 (River sediments, Aquatic, Spain)
Malbranchea californiensis ATCC 15600 T / NR 121259.1
Paracoccidioides brasiliensis UAMH 8037 T / AF038360.1
Blastomyces percursus CBS 139878 T / NR 153647.1
0.02

Figure S5. RAxML phylogenetic tree showing the relationships among some species in the *Malbrancheaceae*, and related ITS1/ITS2 environmental sequences deposited in the GlobalFungi data base. Titles of sequences contain sequence and sample codes taken from GlobalFungi. ITS1/ITS2 sequences of novel *Malbranchea* species are in red. ITS1/ITS2 sequences of sediment isolates and ex-type strains of known species identified in this study are in green. ITS1/ITS2 sequences of known species retreived from GenBank are in bold. Geographical and ecological information contained in environmental sequences associtated to the novel taxa are written in bold. Determined by MEGA software v.6., the best nucleotide substitution model for ML analysis was K2+G for ITS1 and T92+G for ITS2. The alignments data sets were 315 bp long for ITS1 and 149 bp for ITS2; with 148 variable sites for ITS1 and 77 for ITS2; and 134 phylogenetically informative for ITS1 and 66 for ITS2. Branch lengths are proportional to phylogenetic distances. Bootstrap support values above 50% are indicated on the nodes. The trees were rooted to *Paracocciodioides brasiliensis* UAMH 8037 and *Blastomyces percursus* CBS 139878. ^{T}= Ex-type strain.
